# M2 macrophage-derived cathepsin S promotes peripheral nerve regeneration via fibroblast–Schwann cell-signaling relay

**DOI:** 10.1186/s12974-023-02943-2

**Published:** 2023-11-09

**Authors:** Eri Oshima, Yoshinori Hayashi, Zhen Xie, Hitoshi Sato, Suzuro Hitomi, Ikuko Shibuta, Kentaro Urata, Junjun Ni, Koichi Iwata, Tatsuo Shirota, Masamichi Shinoda

**Affiliations:** 1 Department of Oral and Maxillofacial Surgery, Showa University School of Dentistry, 2-1-1 Kitasenzoku, Ota-ku, Tokyo, 142-8515 Japan; 2 Department of Physiology, Nihon University School of Dentistry, 1-8-13, Kandasurugadai, Chiyoda-Ku, Tokyo, 101-8310 Japan; 3 Key Laboratory of Molecular Medicine and Biotherapy in the Ministry of Industry and Information Technology, Department of Biology, School of Life Science, Beijing Institute of Technology, Beijing, 100081 China; 4 Department of Complete Denture Prosthodontics, Nihon University School of Dentistry, 1-8-13, Kandasurugadai, Chiyoda-Ku, Tokyo, 101-8310 Japan

**Keywords:** Macrophage, Schwann cells, Ephrin-B2, Cathepsin S, Peripheral nerve injury, Axon regeneration, Gene expression

## Abstract

**Background:**

Although peripheral nerves have an intrinsic self-repair capacity following damage, functional recovery is limited in patients. It is a well-established fact that macrophages accumulate at the site of injury. Numerous studies indicate that the phenotypic shift from M1 macrophage to M2 macrophage plays a crucial role in the process of axon regeneration. This polarity change is observed exclusively in peripheral macrophages but not in microglia and CNS macrophages. However, the molecular basis of axonal regeneration by M2 macrophage is not yet fully understood. Herein, we aimed to identify the M2 macrophage-derived axon regeneration factor.

**Methods:**

We established a peripheral nerve injury model by transection of the inferior alveolar nerve (IANX) in Sprague–Dawley rats. Transcriptome analysis was performed on the injured nerve. Recovery from sensory deficits in the mandibular region and histological reconnection of IAN after IANX were assessed in rats with macrophage depletion by clodronate. We investigated the effects of adoptive transfer of M2 macrophages or M2-derived cathepsin S (CTSS) on the sensory deficit. CTSS initiating signaling was explored by western blot analysis in IANX rats and immunohistochemistry in co-culture of primary fibroblasts and Schwann cells (SCs).

**Results:**

Transcriptome analysis revealed that CTSS, a macrophage-selective lysosomal protease, was upregulated in the IAN after its injury. Spontaneous but partial recovery from a sensory deficit in the mandibular region after IANX was abrogated by macrophage ablation at the injured site. In addition, a robust induction of c-Jun, a marker of the repair-supportive phenotype of SCs, after IANX was abolished by macrophage ablation. As in transcriptome analysis, CTSS was upregulated at the injured IAN than in the intact IAN. Endogenous recovery from hypoesthesia was facilitated by supplementation of CTSS but delayed by pharmacological inhibition or genetic silencing of CTSS at the injured site. Adoptive transfer of M2-polarized macrophages at this site facilitated sensory recovery dependent on CTSS in macrophages. Post-IANX, CTSS caused the cleavage of Ephrin-B2 in fibroblasts, which, in turn, bound EphB2 in SCs. CTSS-induced Ephrin-B2 cleavage was also observed in human sensory nerves. Inhibition of CTSS-induced Ephrin-B2 signaling suppressed c-Jun induction in SCs and sensory recovery.

**Conclusions:**

These results suggest that M2 macrophage-derived CTSS contributes to axon regeneration by activating SCs via Ephrin-B2 shedding from fibroblasts.

**Supplementary Information:**

The online version contains supplementary material available at 10.1186/s12974-023-02943-2.

## Introduction

Peripheral nerves have an intrinsic self-repair capacity and repair spontaneously following damage; however, functional recovery is slow and limited [1]. The endogenous recovery of sensory and motor function after nerve injury is unsatisfactory in the clinical setting [2]. In the orofacial region, nerve damage can be seen in the inferior alveolar nerve (IAN) and mental nerve due to dental treatment, surgery, or trauma, leading to a sensory disturbance, such as hypoesthesia or anesthesia in the mandibular region [3, 4]. Sensory disorder worsens patients’ daily lives, including eating and talking, for several months to years [5]. Although peripheral nerves can repair themselves to some extent, the recovery of their normal function is often incomplete. Therefore, elucidating the mechanisms underlying sustained axon regeneration is important for developing therapeutic strategies following axon damage.

An increasing number of animal studies indicate that macrophages and Schwann cells (SCs) are essential in axon regeneration after peripheral nerve injury [6, 7]. It has been shown that peripheral macrophages can be divided into the “classically activated” pro-inflammatory phenotype (M1) and “alternatively activated” anti-inflammatory phenotype (M2) [8], distinct from the microglia and CNS macrophages [9]. Flow cytometry analysis clarified the phenotype of macrophages at the injured site of the peripheral nerve. The number of M1 macrophages peaks at the injury site 1–2 days after injury and then decreases afterward. On the other hand, the number of M2 macrophages reached its highest level on the third day after injury and remains constant after that [10]. M2-polarized macrophages promote axon regeneration [11, 12]. Furthermore, macrophage depletion by administering ganciclovir in CD11b-TK^mt−30^ mice or clodronate liposomes in wild-type mice disrupts axon regeneration following sciatic nerve injury [13–15]. In the case of SCs, after axon damage, SCs acquire repair-supportive phenotypes. These phenotypes involve the production of neurotrophic factors [16–18], secretion of several cytokines that accelerate immune responses [19], facilitation of remyelination [20], and formation of regeneration tracks called Büngner bands [21], which accelerate the extension of an axon toward the target site. Furthermore, SCs eliminate myelin debris, which inhibits axon outgrowth after peripheral nerve injury [21]. Accordingly, macrophages and SCs play critical roles in axon regeneration and rapidly create a pro-regenerative environment [6, 7]. Signal relay from macrophages to SCs has also been shown to contribute to the conversion of SCs to a repair-supportive phenotype and consequently promote axon regeneration [22, 23]. Thus, intercellular signaling at the injured site plays an important role in axon regeneration.

The limited regeneration capacity of damaged axons could be attributed to various reasons. Although SCs transform into a repair-supportive phenotype after peripheral nerve injury, repair-supportive SCs are not maintained at the injured site for long periods, and c-Jun disappears rapidly after 10 weeks [24, 25]. Macrophages are also not present for long periods at the site of injury. They accumulate at the injury site, polarize into an anti-inflammatory M2 phenotype [10], and eventually egress over time before completing axon regeneration [10]. Accordingly, the sustained activity of repair-supportive SCs and M2 macrophages at the peripheral nerve injury site may lead to long-lasting and effective axon regeneration. c-Jun expression in SCs has been identified to be responsible for preserving long-term axon regeneration [24]. These results show that cell-to-cell communication between macrophages and SCs is being elucidated; however, it remains unclear how macrophage-derived molecules contribute to c-Jun expression in SCs during axon regeneration.

Fibroblasts as well as macrophages regulate repair-supportive SCs [26]. Fibroblasts directly contact SCs through Ephrin-B2, transforming SCs into a repair-supportive phenotype [26]. Ephrin-B2 is a class B ephrin and is a ligand for the EphB receptor tyrosine kinase. It is an important regulator of growth and patterning processes in many organs and species, directing axons to their proper targets [27]. EphB2 signaling induces the directional sand collective migration of SCs and axonal regeneration across the cleavage site [26]. Both fibroblast–SC and macrophage–SC signal relays are involved in axon regeneration after damage, although it is unclear whether these intercellular signal relays comprise a series or are independent.

Cathepsin S (CTSS: EC 3.4.22.27) is a lysosomal cysteine protease predominantly expressed in mononuclear phagocytic cells [28]. It has unique properties, causing proteolysis even in the extracellular space at neutral pH [29, 30]. Ephrin B2, a membrane-bound protein, is cleaved into short peptides by extracellular proteases [31], possibly implicating CTSS in the cleavage of Ephrin B2.

Here, we aimed to investigate the mechanisms underlying macrophage–SC crosstalk during axon regeneration after IAN transection (IANX) in rats. We identified that macrophage-derived CTSS plays a key role in axon regeneration by cleaving Ephrin-B2 on fibroblasts, which sequentially activate SCs. CTSS-mediated Ephrin-B2 cleavage was also observed in human sensory nerves. These results suggest that CTSS-mediated therapy may be a promising and effective treatment for axon damage following nerve injury.

## Methods

### Animals

Male Sprague–Dawley rats (6–7 weeks, 160–180 g) were purchased from Japan SLC and maintained in specific pathogen-free conditions at 23 ± 1 °C on a 12 h light/dark cycle, provided food and water ad libitum, and acclimated to the experimenter and experimental environment for at least 7 days. All experiments were performed following the guidelines issued by the animal care and use committee of Nihon University (Protocol number: AP20DEN022). The number of animals used for the experiments was minimized following the 3Rs principle.

### Surgical procedure

All surgical procedures were performed under anesthesia with isoflurane inhalation (induction: 4%; maintenance: 2%). The left cheek skin and masseter muscle were incised approximately 15 mm, and the periosteum was detached to expose the mandibular cortical bone. The surface of cortical bone was removed using a sterilized steel round bar (diameter: 1 mm) to reveal the IAN and mental nerve. For convenience, IANX indicated the transfection of both IAN and mental nerve. IANX was performed by making a 1 mm gap between the proximal and distal stump using micro scissors. The proximal and distal segments were not sutured. Finally, the wound was sutured with a 5–0 silk. In sham rats, the left cheek skin and masseter muscle were incised without IANX. Body temperature was maintained during all surgical procedures. Each experimental schedule was drawn in Additional file 1 (Fig. S1).

### Drug administration at the IANX site

A biodegradable gelatin hydrogel sheet (MedGel PI5; MedGel, Osaka, Japan) was soaked with 100 µL of drugs following the manufacturer’s procedure and applied onto the site of the IAN injury under 2% isoflurane inhalation. Clophosome-A (Clo: 700 µg/100 µL, F70101C-A, FormuMax Scientific, CA, USA), control liposome (Lipo: 100 µL, F70101-A, FormuMax Scientific), saline (100 µL), recombinant human CTSS (rhCTSS: 0.2 µg/100 µL in saline, 1183-CY-010, R&D systems, MN, USA), rhCTSS + Clo, or rhCTSS + Ephrin-B2 blocking peptide (EBP; GTX88049-PEP, Genetex, CA, USA [rhCTSS: 0.2 µg + EBP: 10 µg/100 µL in saline]) were applied 2 day post-IANX. Z-FL-COCHO (Z-FL, a CTSS-specific inhibitor: 10 pmol/100 μL in the vehicle, AOB13823, AOBIOUS, MA, USA) or vehicle were administered 2 and 8 day post-IANX. DMSO (0.05%) in saline was used as the vehicle. Small interference RNA (siRNA) for Ctss (siCtss: 20 pmol/100 µL, s132283, Thermo Fisher Scientific, MA, USA) or negative control siRNA (siCont: 20 pmol/100 µL, 4390843, Thermo Fisher Scientific) were applied 2 and 6 day post-IANX. siRNA was diluted in Lipofectamine RNAiMAX (Thermo Fisher Scientific) at the rate of 3:1 following the manufacturer’s procedure.

### Injection of retrograde neurotracer

Three days before perfusion with 4% paraformaldehyde (PFA), 1 µL of Fluoro-Gold (FG; 4%, Fluorochrome) was injected in the area, where the mental nerve, a terminal branch of IAN, emerged from the mental foramen using a 27-gauge needle under anesthesia with 2% isoflurane inhalation.

### Cell culture

i. Macrophages

Under 2% isoflurane inhalation, ice-cold phosphate-buffered saline (PBS) was injected into the abdominal cavity in naïve rats. The abdomen was rubbed lightly for 10–30 s, and then, the PBS in the abdominal cavity was collected. The isolated cells were treated with RBC lysis buffer (Invitrogen) following the manufacturer’s procedure. Resultant cells were seeded in culture dishes in Dulbecco’s Modified Eagle Medium (DMEM, Nissui, Tokyo, Japan) supplemented with 10% fetal bovine serum (FBS; Nichirei, Tokyo, Japan), penicillin (100 U/mL, Thermo Fisher Scientific), streptomycin (100 μg/mL, Thermo Fisher Scientific), and l-glutamine (2 mmol/L, Fujifilm Wako Pure Chemical, Osaka, Japan) at 37 °C in 5% CO_2_. Two hours later, the dish was washed using PBS 3–4 times to eliminate nonadherent cells.

For the immunohistochemical analysis, macrophages (5 × 10^5^ cells) were seeded onto 13φ glasses (Matsunami, Tokyo, Japan) in a 24-well plate. After 48 h in culture, cells were fixed using 4% paraformaldehyde (PFA) for 1 h to check the purity of macrophages. To analyze the polarization state of macrophages, we stimulated them with IL-4 (100 ng/mL, 204-IL, R&D systems) and IL-13 (100 ng/mL, 200–13, PeproTech, NJ, USA) or lipopolysaccharide (100 ng/mL, L2630, Sigma-Aldrich, MO, USA) and interferon-γ (100 ng/mL, R&D systems) for 48 h. Subsequently, macrophages were fixed using 4% PFA.

For western blot analyses, macrophages (1.2 × 10^6^ cells) were seeded in a 6-well plate and treated with IL-4/IL-13 or saline for 48 h. The supernatant and cells were collected. The specimens were stored at − 80 °C until use.

For the adoptive transfer of macrophages, we used IL-4/IL-13 stimulated or siRNA-treated IL-4/IL-13-stimulated macrophage. Macrophages (5 × 10^6^ cells) were detached from the culture plate with 0.05% trypsin/EDTA (Thermo Fisher Scientific) 48 h after IL-4/IL-13 stimulation and resuspended with DMEM.

For CTSS knockdown, macrophages (5 × 10^6^ cells) were treated with siCtss (10 nM) or siCont (10 nM) during IL-4/IL-13 stimulation.

ii. Trigeminal ganglion (TG) neurons

TGs were dissected from neonatal rats (postnatal days 1–5) and were incubated with HBSS containing collagenase II (2 mg/mL, Worthington, NJ, USA) and papain (20 U/mL, Worthington) for 55 min at 37 °C in 5% CO_2_, followed by the addition of DNase (10 U/mL, Worthington) for 5 min. The reaction was stopped by adding FBS containing DMEM. Cells were washed 3 times with 1 mL DMEM. A two-compartment microfluidic device (Xona Microfluidics, NC, USA) was placed on 0.01% poly-l-lysine (Sigma-Aldrich) coated glass, and cells (1 × 10^5^ cells/10 μL in DMEM) were seeded in the soma compartment which was supplemented with nerve growth factor (100 ng/mL, Sigma-Aldrich) and Glial cell-derived neurotrophic factor (10 ng/mL, PeproTech) to sustain neuronal survival. Next, 5-fluorouracil (1 μM, Sigma-Aldrich) was added to the medium to eliminate proliferating cells. After 7 days in culture, CTSS (final concentration: 50 nM) or saline was added to the axon compartment. Cells were fixed using 4% PFA 24 h after CTSS treatment.

iii. Co-culture of SCs and fibroblasts

The trigeminal nerves were dissected from neonatal rats and treated with enzymes containing HBSS according to the same method described above section. Cells (2 × 10^6^ cells) were suspended in Eagle’s Minimum Essential Medium (MEM, Nissui) supplemented with 0.17% NaHCO_3_, 0.2% glucose, 10% FBS, L-glutamine (2 mmol/L), penicillin (100 U/mL), and streptomycin (100 µg/mL) and seeded on a 6-well plate coated with laminin (10 µg/mL, Corning, NY, USA). SCs were isolated from mixed culture according to the previous methods [32, 33]. After 2 days in culture, the culture medium was replaced with collagenase NB4 (0.07 U/mL, Nordmark, Uetersen, Germany)-containing serum-free MEM for 30 min at 37 °C to isolate SCs, and isolated SCs were resuspended with serum-containing MEM. Following centrifugation at 1000 × g for 5 min, cells were treated with Thy-1.1 antibody (1:1000, HIS51, eBioscience, CA, USA) containing MEM for 30 min at 4 °C. The supernatant was discarded after centrifugation, and cells were treated with Class I Complement (1:10, One Lambda, CA, USA) containing MEM for 45 min at 37 °C to lysis fibroblasts. Then, SCs (2 × 10^5^ cells) were seeded onto laminin-coated 13φ glasses in a 24-well plate. After 2 days in culture, the cells (fibroblasts) remaining in the 6-well culture plate were isolated with 0.05% trypsin–EDTA. Then, cells (2 × 10^5^ cells) were seeded onto cell culture inserts with a pore diameter of 3 µm (Falcon) and placed in a 24-well plate seeded with SCs. Co-cultured cells were treated with rhCTSS (50 nM), rhCTSS + Z-FL (2 μM), or rhCTSS + EBP (10 µg/mL). SCs were fixed with 4% PFA 24 h after drug treatment. To evaluate the purity of isolated SCs and fibroblasts, we plated these cells on laminin-coated 13φ glasses in a 24-well plate and then fixed them with 4% PFA. Immunohistochemical analyses were performed as described in the Immunohistochemistry sections.

### Adoptive transfer of macrophages

M2 macrophages or siRNA-treated M2 macrophages were resuspended in DMEM. M2 macrophages (2 × 10^5^ cells/50 µL in DMEM) were injected at the site of the IAN, then MedGel soaked with Z-FL (10 pmol/50 µL) or vehicle (50 µL) was applied onto the injured IAN similar to the procedure described above. DMSO (0.05%) in saline was used as the vehicle.

### Mechanical sensitivity assay

Mechanical sensitivity of the lower lip post-IANX was assessed using digital forceps (Bioseb, Vitrolles, France). First, the rats were anesthetized with 2% isoflurane inhalation. After stopping the isoflurane supply, a weak anesthesia level was confirmed by pinching a hindlimb with a stimulus intensity of 80–130 g. When the rats exhibited a hindlimb flexion reflex by pinching, mechanical stimulation was applied to the left lower lip. When the rats regained consciousness and began to move spontaneously, weak anesthesia was performed again. Mechanical stimulation was applied to the left lower lip with an increment of mechanical intensity (10 g/s). The cutoff value was set as 200 g to avoid tissue damage. The experimenter immediately stopped pinching when the rats started to show a head withdrawal response. The HWT was determined when the rats exhibited a head withdrawal response against pinching by digital forceps. The HWT was measured three times, followed by calculating the average value. Mechanical sensitivity tests were performed before surgery and every other day until postoperative days 8 or 14.

### Collection of a sensory nerve from patients

The human study was approved by the Institutional Ethics Committee of Showa University (Protocol No. 22-282-A) and conducted following the principles of the Helsinki Declaration. Four patients (males: 35 and 21 years, female: 32 and 40 years) underwent Le Fort I osteotomy at the Department of Oral and Maxillofacial Surgery of Showa University Dental Hospital, Japan. Preoperative radiographic evaluation, clinical examination, and planning were identical for all patients. Informed written consent was obtained from each patient after they received an explanation regarding treatment. A part of the nasopalatine nerve transected bluntly during down fracture was removed as a discarded biological specimen from patients who underwent Le Fort I osteotomy. The excised nasopalatine nerve was placed in 4% PFA overnight at 4 °C, followed by incubation in 20% sucrose in PBS. For the western blot analysis, the specimens were placed on dry ice immediately after collection.

### Immunohistochemistry

After terminating the mechanical sensitivity assay, the rats were transcardially perfused with ice-cold saline, followed by 4% PFA in 0.1 M phosphate buffer (4 °C, pH 7.4) under isoflurane (5%) anesthesia. The IAN segments range from proximal to distal from the injured site, and the TGs were collected. Excised tissues were post-fixed in 4% PFA overnight at 4 °C and subsequently were allowed to equilibrate in 20% sucrose in PBS overnight at 4 °C for cryoprotection.

Slices (11 μm-thickness) of the IANs, TGs, or nasopalatine nerves were made using a cryostat (Tissue-Tek Polar, Sakura Finetek, Tokyo, Japan) and mounted on the MAS-coat glass (Matsunami). After washing with PBS 3 times for 10 min, the sections were incubated with PBS containing 4% normal donkey serum and 0.3% Triton X-100 for 1 h at room temperature. Then, these sections were incubated with primary antibodies for IBA1 (goat polyclonal, 1:1000, ab5076, Abcam, Cambridge, UK; rabbit polyclonal, 1:1000, 019-19741, Fujifilm Wako Pure Chemical), c-Jun (rabbit polyclonal, 1:500, 9165, Cell-Signaling Technology, MA, USA), S100β (mouse polyclonal, 1:1000, ab4066, Abcam; rabbit polyclonal, 1:1000, ab76729, Abcam), αSMA (mouse polyclonal, 1:500, A5228, Sigma-Aldrich), NF200 (chicken polyclonal, 1:1000, ab4680, Abcam), CTSS (goat polyclonal, 1:50, ab18822, Abcam), CD206 (rabbit polyclonal, 1:200, ab64693, Abcam), CD11c (mouse polyclonal, 1:250, ab11029, Abcam), Ephrin-B2 (rabbit polyclonal, 1:50, HPA008999, Sigma-Aldrich), or EphB2 (mouse polyclonal, 1:1000, AF467, R&D systems) in PBS overnight at 4 °C.

For the staining of primary cultured macrophages, TG neurons, SCs, and fibroblasts, fixed cells were washed with PBS three times for 10 min. After blocking, cells were incubated with IBA1 (1:2000), CD206 (1:500, Abcam), β3-Tubline (mouse monoclonal, 1:1000, MAB1195, R&D systems), S100β (1:500, Abcam), αSMA (1:1000, Sigma-Aldrich), or c-Jun (1:1000, Cell Signaling) overnight at 4 °C.

The slices or cells were further incubated with secondary antibodies conjugated with Alexa Fluor 488 or 555 (1:500 in each, Thermo Fisher Scientific) for 2 h at room temperature. Hoechst-33258 (1:1000, 14530, Sigma-Aldrich) was used to visualize the nucleus of all cell types. The TG sections from FG-injected rats were incubated with NeuroTrace 530/615 red fluorescent Nissl stain (1:1000, N21482, Thermo Fisher Scientific) for 20 min at room temperature. The specimens were mounted in an antifading medium (PermaFluor Aqueous Mounting Medium, Thermo Fisher Scientific). Images were captured using a BZ-X800 (Keyence, Osaka, Japan) or a Andor BC43 benchtop confocal microscope (Andor Technology, Belfast, UK) with Imaris Viewer software (Oxford Instruments, Abingdon, UK).

The area occupied by IBA1 immunofluorescence and the number of c-Jun-positive cells at the injured site of the IAN was assessed within the region of interest (ROI) that was determined by enclosing the edge of the IAN in the image with the polygon selection tool using ImageJ software (NIH, http://rsbweb.nih.gov/ij/). These images were binarized followed by measuring the area occupied by IBA1 immunofluorescence within ROI. The number of c-Jun-positive cells within ROI was counted and calculated as the number of cells in 10,000 μm^2^. FG-labeled TG neurons in TG and IBA1-positive cells in cultured macrophages in images were counted using the ImageJ plugin Cell count, and the count was normalized by the total number of TG neurons stained by Nissl or the total number of Hoechst-33258-positive cells. Five slices were stained per animal, followed by calculating the average value.

Axons of primary TG neurons visualized with anti-β3-Tubulin antibody were traced manually, where they emerged from the microgroove on the axon compartment side, and their lengths were measured using Image J software. Three axons per section were randomly selected, and the average axon length was calculated.

### Western blotting

After terminating the mechanical sensitivity assay, the rats were transcardially perfused with ice-cold saline under isoflurane (5%) anesthesia, and then the IANs were immediately excised. Cultured macrophages were collected as described above. The specimens were stored at − 80 °C until use. The IANs or cultured macrophages were homogenized in lysis buffer (10 mM Tris–HCl [pH 7.4], 150 mM NaCl, 1% Triton X-100, and 0.5% NP-40) with 1% protease inhibitors (Sigma-Aldrich). The protein concentrations were measured using a BCA assay.

For the analysis of band shift of Ephrin-B2, recombinant human Ephrin-B2 (1 μg, 7397-EB, R&D systems) was mixed with rhCTSS (0.25 μg) in reaction buffer (50 mM sodium acetate [pH 5.5] and 4.0 mM dithiothreitol) in microtube for 30 min at 37 °C in the presence or absence of Z-FL (20 μM).

The human nasopalatine nerves were homogenized in a lysis buffer without protease inhibitors. The protein concentrations were measured using a BCA assay kit. The lysate (protein concentration: 2.5 μg) was mixed with rhCTSS (0.2 μg) in the reaction buffer for 30 min at 37 °C in the presence or absence of Z-FL (20 μM or 200 μM). The components of both lysis and reaction buffers are described above.

An equal amount of protein (2.5–15 μg) or 14 μL of supernatant from macrophage culture was mixed in Laemmli sample buffer with 2-mercaptoethanol, which in turn was heat-denatured. Proteins were separated by 10% sodium dodecyl sulfate–polyacrylamide gel electrophoresis (Bio-Rad, CA, USA). Gels were transferred onto Amersham Hybond-P 0.45 polyvinylidene difluoride membrane (Cytiva, MA, USA). Membranes were blocked with TBS-T (0.2% Tween-20 diluted in Tris-buffered saline) containing 5% Blocking-One (Nacalai Tesque, Kyoto, Japan) for 1 h at room temperature and then incubated overnight at 4 °C with primary antibodies for anti-Ephrin-B2 (1:1000, Sigma-Aldrich), CTSS (1:500, Abcam), CD206 (1:1000, Abcam), CD11c (1:1000, Abcam), or β-actin (mouse monoclonal, 1:5000, Abcam, ab8226). After washing with TBS-T 4 times for 5 min, membranes were incubated with horseradish peroxidase-conjugated anti-rabbit, anti-goat, or anti-mouse secondary antibodies (1:1000 in each; NA934V or NA931VS, Cytiva or HAF109, R&D systems) for 2 h at room temperature. The bands were visualized using an image analyzer (Amersham Image Quant 800, Cytiva) after reaction in Western Lightning ELC Pro (PerkinElmer, MA, USA) or Immobilon (Millipore, MA, USA). The band intensity was quantified using ImageJ and normalized to β-actin expression.

### RNA extraction, library preparation, RNA sequencing, and transcriptome analysis

Fourteen days after surgery, the IAN was excised from sham and IANX rats (three biological replicates in each) and immediately immersed into TRIsure reagent (Nippon Genetics, Tokyo, Japan) for RNA extraction following the manufacturer’s protocol. The RNA-containing layer was separated using chloroform, and total RNA was precipitated using a precipitation carrier (Dr. GenTLE, Takara, Shiga, Japan). The quality of RNA was assessed by Agilent Bioanalyzer 2100 (Agilent Technologies, CA, USA). RNA-seq experiments were performed by Novogene (Beijing, China). mRNA was purified from total RNA using poly-T oligo-attached magnetic beads. After fragmentation, mRNA was transcribed into cDNA using NEBNext Ultra RNA Library Prep Kit for Illumina (New England Biolabs). cDNA was processed to 3′ tail adenylation and adapter ligation. RNA-seq was performed on the Illumina NovaSeq 6000 platform (NVCS v1.7.5/RTA v3.4.4) with 2 × 150 bp paired-end sequencing. De-multiplexing and conversion to FASTQ format were performed using the bcl2fastq software (v2.19). The annotation files for the reference genome and gene models were downloaded directly from the genome website. The reference genome index was built using Hisat2 (v2.05), and paired-end clean reads were aligned to the reference genome using Hisat2 (v2.05). The mapped reads of each sample were assembled by StringTie (v1.3.3b) in a reference-based approach for the prediction of novel transcripts [34]. The program featureCounts (v1.5.0-p3) was used to count the read numbers mapped to each gene. Differential expression analysis of two groups (three biological replicates for each group) was performed using the DESeq2 R package (v1.20.0). The *p* values were adjusted using Benjamini and Hochberg’s approach for controlling the false discovery rate. Genes with an adjusted *p* value (padj) < 0.05 found by DEseq2 were assigned as differentially expressed. Genes abundant in macrophages were selected from RNA-seq data with padj changes less than 0.05 and fold changes > twofold using the Harmonizome database [35]. Gene Ontology (GO) enrichment analysis of selected genes was performed using the Metascape database platform to enrich and analyze [36]. Raw data can be accessed on Gene Expression Omnibus (GEO) under the accession number GSE232487.

### Statistics

No data points were removed from the statistical analysis. Drug selection, adoptive transfer, mechanical sensitivity assay, immunohistochemistry, western blot, and statistical analyses were performed separately and in a blinded manner. Data normality was assessed using the Shapiro–Wilk test. Data are presented as the median ± interquartile range or mean ± standard error of the mean (SEM) depending on data normality. Statistical analyses were performed using one-way analysis of variance (ANOVA) post hoc Tukey’s test, Kruskal–Wallis post hoc Dunn’s test, unpaired *t* test, Mann–Whitney *U* test, or Friedman test post hoc Dunn’s test (a repeated measure of mechanical sensitivity assay within the group) using GraphPad Prism 9 (GraphPad Software, CA, USA). Repeated measurements of mechanical sensitivity assay between the groups were assessed using the generalized estimating equations (GEE) method post hoc Bonferroni’s test in SPSS version 28 (IBM, NY, USA). Differences were considered significant at *P* < 0.05.

## Results

### Macrophage depletion at the injury site inhibits peripheral nerve regeneration

**Fig. 1:**
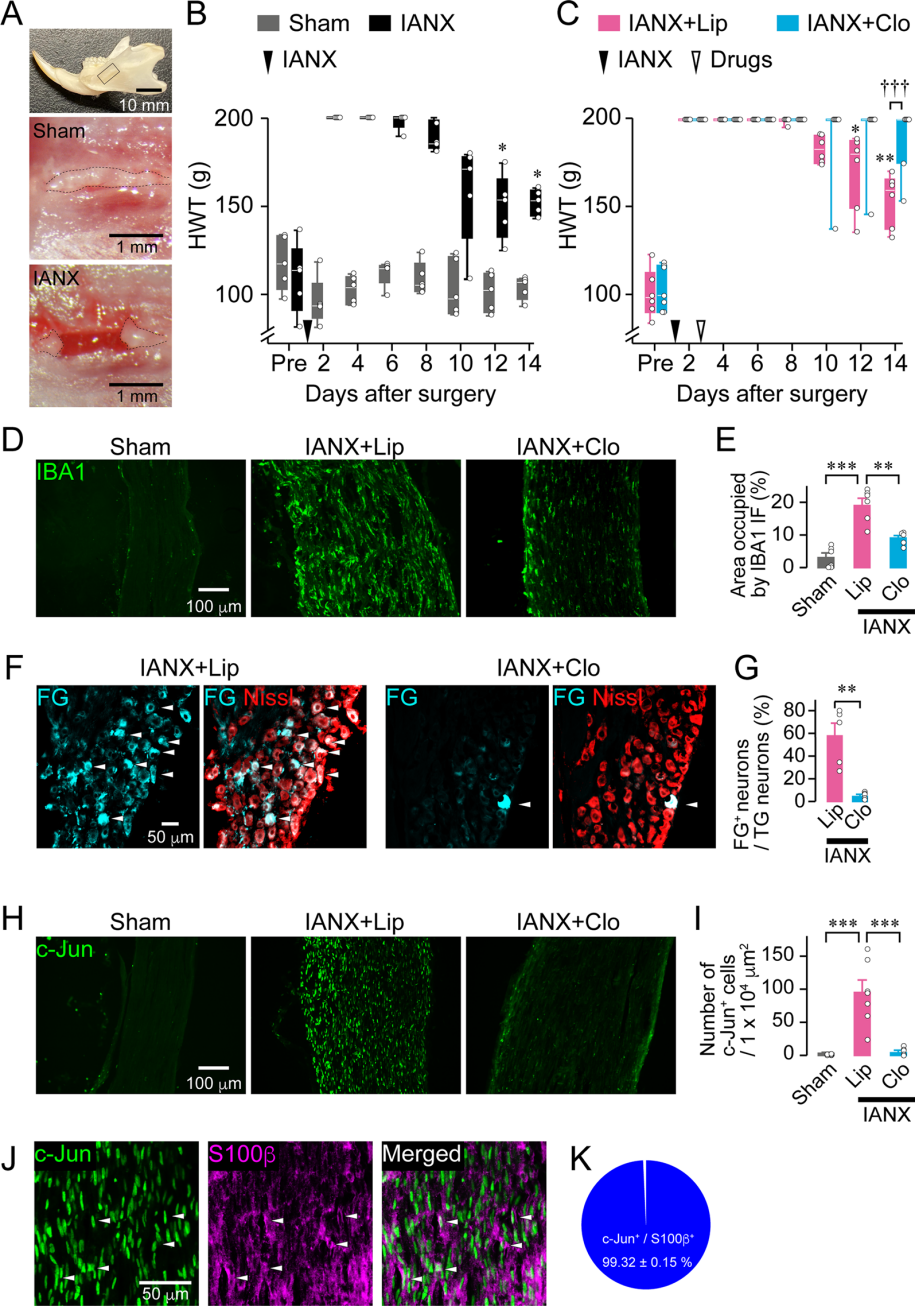
Macrophages contribute to sensory recovery in the lower lip after inferior alveolar nerve (IAN) transection. **A** Photographs showing the rat mandibular region (top) and IAN in sham rats (middle) and inferior alveolar nerve-transected (IANX) rats (bottom). The IAN is located under the bone in the area enclosed by the solid line (top). **B** Time course of head withdrawal threshold (HWT) of sham and IANX rats post-surgery. *n* = 5, Friedman test post hoc Dunn’s test, *P < 0.05 vs. day 2. **C** Time course of HWT of IANX rats that were administered with control liposome (Lipo) or clodronate liposome (Clo). IANX + Lipo (*n* = 6); IANX + Clo (*n* = 7), Friedman test post hoc Dunn’s test, **P < 0.01 vs. day 2; GEE post hoc Bonferroni’s test. **D** Images showing IBA1 immunofluorescence at the site of IAN injury 14 day post-IANX. **E** Columns represent the average values of the area occupied by IBA1 immunofluorescence at the injured site. *n* = 5–6, one-way ANOVA post hoc Tukey’s test. **P < 0.01, ***P < 0.001. **F** Images showing Fluoro-Gold (FG)-labeled trigeminal ganglion (TG) neurons at 14 day post-IANX. **G** Columns represent the average number of FG-labeled TG neurons out of the total TG neurons. *n* = 5 in each, unpaired *t* test. **P < 0.01, ***P < 0.001. **H** Images showing c-Jun immunofluorescence at the injured site 14 day post-IANX. **I** Columns represent the average number of c-Jun-positive cells at the injured site. *n* = 5–7, one-way ANOVA post hoc Tukey’s test. ***P < 0.001. **J** Images showing c-Jun and S100β immunofluorescence at the injured site 14 day post-IANX. **K** Pie chart showing the percentage of c-Jun-positive cells out of S100β-positive cells at the injured site 14 day post-IANX. *n* = 5

To investigate axon regeneration after peripheral nerve injury, we transected IAN, the third branch of the trigeminal nerve conveying somatosensory information in the mandibular region to the central nervous system, in rats (Fig. 1A). Head withdrawal threshold (HWT) was measured as mechanical sensitivity upon pinching of lower lips in rats. For 4 days, HWT showed the cutoff value (200 g), and a significant reduction from the cutoff value was observed 12th day onward post-IANX (Friedman test post hoc Dunn’s multiple comparison test, *P* < 0.0001, vs. day 2; Fig. 1B). Conversely, HWT in sham rats remained unchanged throughout the measurement period (Friedman test post hoc Dunn’s multiple comparison test, *P* = 0.2432, vs. Pre; Fig. 1B). These results suggest that IANX causes a temporal sensory deficit in the mandibular region partially recovered from hypoesthesia due to an endogenous regenerative capacity.

The effects of macrophages on recovery from hypoesthesia post-IANX were examined by locally administering Clo to deplete macrophages or Lipo at the injury site of IAN. In Lipo-administered rats, HWT showed temporary sensory deficits for 10 day post-IANX and subsequent spontaneous recovery from hypoesthesia (Friedman test post hoc Dunn’s multiple comparison test, *P* < 0.0001, vs. day 2; Fig. 1C), similar to that in IANX-subjected rats. In Clo-administered rats, HWT remained at the cutoff value throughout the experimental period (Friedman test post hoc Dunn’s multiple comparison test, *P* = 0.298, vs. day 2; Fig. 1C). A significant difference in HWT was observed between these groups at 14 day post-IANX (GEE post hoc Bonferroni’s test, *P* = 0.004, df = 1, Wald χ^2^ = 8.256, IANX + Lipo vs. IANX + Clo; Fig. 1C). To confirm macrophage depletion caused by Clo administration, we stained the injury site 14 day post-IANX with anti-IBA1 antibody. The area occupied by IBA1 immunofluorescence at the injured site in Clo-administered rats was significantly less than that in Lipo-administered rats (IANX + Lipo: 19.20% ± 2.07%; IANX + Clo: 9.15% ± 0.88%, one-way ANOVA post hoc Tukey’s test, *P* < 0.0001, F_(2,14)_ = 27.31; Fig. 1D, E). In the intact IAN of sham rats, a degree of IBA1 immunoreactivity was observed (sham: 3.26% ± 1.36%; Fig. 1D, E). The anatomical continuity between the proximal and distal stumps of IAN was evaluated when rats regained sensory function on day 14 post-IANX. At the macroscopic level, severed axonal ends reconnected in both Lipo- and Clo-administered rats, with an anatomical continuity comparable to that of the intact IAN in sham rats (Additional file 1: Fig. S2A). In contrast, immunohistochemical analysis showed apparent differences in the injured IAN between Lipo- and Clo-administered rats. In Lipo-administered rats, the fluorescence intensity of neurofilament 200 (NF200, an axonal marker) in proximal and distal stumps of IAN was almost the same as in sham rats. However, NF200 immunofluorescence in the distal stump of the IAN was faint in Clo-administered rats (Additional file 1: Fig. S2B). To quantify the extent of nerve reconnection, we injected FG (a retrograde neurotracer) in the mental nerve emerging from the mental foramen located ~ 10 mm peripherally from the injured site. We then counted the number of FG-labeled TG neurons in the third branch of the TG. In Lipo- and Clo-administered rats, 57.84% ± 11.14% and 5.28% ± 1.28% of TG neurons were labeled with FG conveyed via axonal transport, respectively (unpaired *t* test, *P* = 0.0016, t_(8)_ = 4.687; Fig. 1F, G). These results indicate that macrophage accumulation at the injured IAN is required for axon regeneration.

The representative transcription factor c-Jun initiates a repair program in SCs, contributing to axon regeneration [7, 17]. To investigate the relationship between macrophages and SCs, we stained the injured site of IAN using an anti-c-Jun antibody after macrophage depletion. Robust c-Jun expression was observed at the injury site in Lipo-administered rats, whereas c-Jun positive cells were hard to detect in the intact IAN of sham rats (sham: 0.65 ± 0.26 cells/1 × 10^4^ μm^2^; IANX + Lipo: 95.58 ± 18.23 cells/1 × 10^4^ μm^2^; one-way ANOVA post hoc Tukey’s test, *P* = 0.0002, F_(2,14)_ = 17.28; Fig. 1H, I). A few c-Jun-positive cells were detected at the injured site, wherein macrophages were depleted after Clo-administration (6.93 ± 2.56 cells/1 × 10^4^ μm^2^). We then evaluated the cell-specific expression pattern of c-Jun at the injured site. The majority of c-Jun expression was observed in S100β-positive cells (SCs: 99.32% ± 0.15%), while minimal expression was observed in IBA1-positive (macrophages: 2.86% ± 0.5%) and αSMA-positive cells (fibroblasts: 0.45% ± 0.5%) (Fig. 1J, K, and Additional file 1: Fig. S3). These results suggest that c-Jun expression in SCs at the injured site of the IAN depends on macrophages.

### M2 macrophage-derived cathepsin S promotes recovery from hypoesthesia in the lower lip post-IANX

**Fig. 2:**
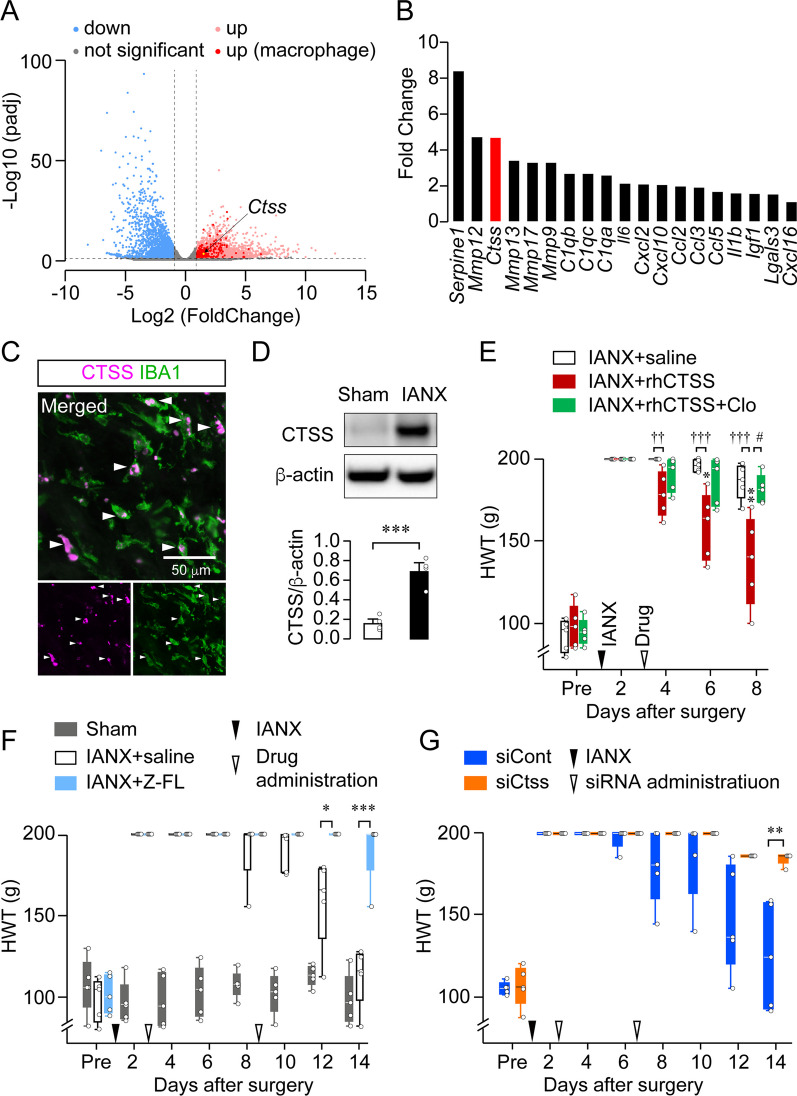
CTSS is required for the sensory recovery of the lower lip post-IANX. **A** Volcanic plot showing the gene expression changes between sham and IANX rats at the site of IAN injury on day 14 post-surgery (*n* = 3). Blue and red dots indicate the downregulated and upregulated genes at the injured site, respectively. Vivid red dots indicate upregulated genes, especially in macrophages. The yellow dot indicates *Ctss* mRNA. **B** Secretory molecules from macrophages among the upregulated genes. **C** Images showing IBA1/CTSS double-positive cells at the injured site 14 day post-IANX. **D** Blot of CTSS at the injured site 14 day post-surgery. The column represents the average values of CTSS/β-actin. *n* = 4 in each, unpaired *t* test. ***P < 0.001. **E** Time course of HWT in IANX rats treated with saline, recombinant human CTSS (rhCTSS), or rhCTSS + Clo. *n* = 5 in each, Friedman test post hoc Dunn’s test, *P < 0.05, **P < 0.01 vs. day 2; GEE post hoc Bonferroni’s test. ^††^*P* < 0.01, ^†††^*P* < 0.001, #*P* < 0.05. **F** Time course of HWT in IANX rats treated with saline or Z-FL and sham rats. *n* = 5 in each, GEE post hoc Bonferroni’s test. *P < 0.05, ***P < 0.001. **G** Time course of HWT in IANX rats treated with negative control siRNA (siCont) or Ctss siRNA (siCtss). *n* = 5 in each, GEE post hoc Bonferroni’s test. **P < 0.01. Boxes show the 25th–75th percentiles with the median value as a line within each box, and whiskers indicate the 10th and 90th percentiles of the data in **E**, **F**, and **G**. All data points are shown as open circles

We collected IANs from sham and IANX rats on postoperative day 14, and bulk RNA-sequencing (RNA-seq) was performed to explore macrophage-derived axon regenerative factors (three biological replicates in each group). Hierarchical clustering of the DEGs from the IAN demonstrated that samples from sham and IANX rats largely segregated with each other (Additional file 1: Fig. S4). Of 21,685 genes analyzed, 2419 and 1944 were upregulated and downregulated post-IANX, respectively (Fig. 2A, Additional file 1: Fig. S4, and Additional file 2). A comparison of the 2,419 upregulated genes (light red-cluster genes) with the Harmonizome database [35] elucidated that 216 genes (vivid red-cluster genes) were overexpressed in macrophages compared to other cell types (Fig. 2A and Additional file 3). Gene Ontology enrichment analyses of these 216 genes in our data set revealed that they were associated with positive regulation of immune response, regulation of defense response, response to lipopolysaccharide, innate immune system, cell activation, regulation of leukocyte activation, positive regulation of cytokine production, regulation of inflammatory response, leukocyte chemotaxis, and negative regulation immune system process (Additional file 1: Fig. S5). Macrophage-secretory factors may mediate intercellular signaling between macrophages and SCs post-IANX, eventually leading to axon regeneration. Of macrophage-specific genes, we extracted macrophage-secreting factors that mediate intercellular signaling between macrophages and SCs. Nineteen genes corresponded to macrophage-secreting factors, such as proteases, complement, cytokines, and chemokines (Fig. 2B). We excluded the top two candidate genes, *Serpine1* and *Mmp12*, for the following reasons. First, it has been reported that *Serpine1* mRNA is expressed in both macrophages and SCs [37], whereas *Mmp12* has been implicated in axon regeneration in the sciatic nerve after its injury [38]. After eliminating these two genes, the most abundant transcript expressed in macrophages was *Ctss*. We thus expected CTSS to be an axon regenerative factor. CTSS (EC 3.4.22.27) is a lysosomal cysteine protease predominantly expressed in mononuclear phagocytic cells [28]. Immunohistochemical analyses revealed that CTSS immunofluorescence exclusively merged with that of IBA1-positive cells at the site of injury 14 day post-IANX (Fig. 2C). Western blot analyses also showed that the amount of CTSS was significantly increased in the IAN of IANX-subjected rats compared with that in sham rats (unpaired *t* test, *P* = 0.0007, t_(6)_ = 6.315; Fig. 2D), consistent with the RNA-seq data.

To explore the effect of CTSS on axon regeneration, we locally administered recombinant human CTSS (rhCTSS) or saline at the site of IAN injury 2 day post-IANX. The rhCTSS-administered rats showed a significant decrease in HWT on day 4 post-IANX compared with saline-administered rats (GEE post hoc Bonferroni’s test, *P* < 0.001, df = 1, Wald χ^2^ = 31.356, IANX + saline vs. IANX + rhCTSS; Fig. 2E). Macrophage-depleted rats showed no recovery of sensory deficits throughout the experimental period (Fig. 1C). However, rhCTSS administration at the injured site caused a significant decrease in HWT in macrophage-depleted rats day from 8 onward post-IANX (Friedman test post hoc Dunn’s multiple comparison test, *P* = 0.0186, vs. day 2; Fig. 2E). In contrast, administration of Z-FL (a CTSS-specific inhibitor) at the injured site significantly delayed the reduction of HWT from the cutoff value (Friedman test post hoc Dunn’s multiple comparison test, *P* = 0.4232 vs. day 2; Fig. 2F). In saline-administered rats, HWT showed a significant decrease from the cutoff value from day 12 onward post-IANX (Friedman test post hoc Dunn’s multiple comparison test, *P* = 0.0003, vs. day 2; Fig. 2F). This result was similar to the time course of HWT post-IANX (Fig. 1B). Statistical differences were observed between the effects of saline and Z-FL administration at 12 and 14 day post-IANX (GEE post hoc Bonferroni’s test, *P* < 0.001, df = 1, Wald χ^2^ = 43.016; Fig. 2F). We also conducted the gene silencing of *Ctss* at the injured site by administering small interfering RNA (siRNA) 2 and 6 day post-IANX. The time course of HWT post-IANX after administering negative control siRNA (siCont) was similar to that of saline administration (Fig. 2F, G). However, CTSS siRNA (siCtss) administration significantly delayed the reduction of HWT from the cutoff value (GEE post hoc Bonferroni’s test, *P* = 0.002, df = 1, Wald χ^2^ = 9.89; Fig. 2G). CTSS knockdown efficiency at the injured site was analyzed using western blot analysis. The expression level of CTSS at this site reduced significantly following siCtss administration compared with that after siCont administration (unpaired *t* test, *P* = 0.0194, t_(8)_ = 2.917; Additional file 1: Fig. S6).

**Fig. 3:**
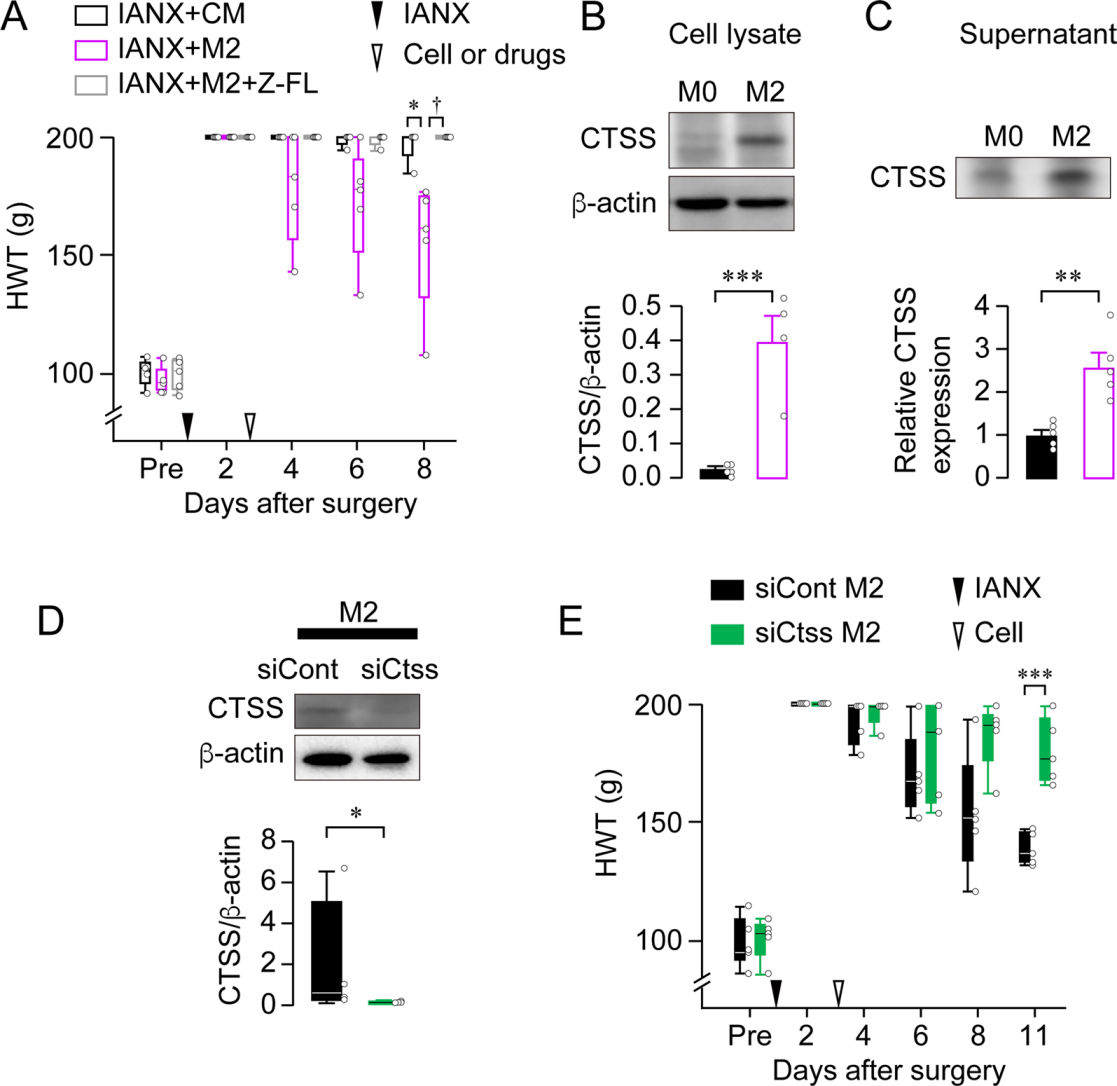
Macrophage-derived CTSS is involved in the sensory recovery of the lower lip post-IANX. **A** Time course of HWT in IANX rats treated with control medium (CM), M2 macrophage (M2), or M2 + Z-FL. *n* = 5 in each, GEE post hoc Bonferroni’s test. *P < 0.05, ^†^*P* < 0.05. **B, C** Blot of CTSS in the cell lysate (**B**) or supernatant (**C**) of non-stimulated macrophages (M0) and IL-4/IL-13-stimulated macrophages (M2). The column represents the average values of CTSS/β-actin. *n* = 4–5 independent cultures, unpaired *t* test, ***P < 0.001 in **B**. The column represents the relative expression of CTSS. *n* = 5 in each, unpaired *t* test **P < 0.01 in **C**. **D** Blot of CTSS in M2 macrophage treated with siCont or siCtss. The column represents the average values of CTSS/β-actin. *n* = 5 independent cultures, Mann–Whitney *U* test. *P < 0.05. **E** Time course of HWT in IANX rats transplanted with siCont-treated M2 macrophages or siCtss-treated M2 macrophages. *n* = 5 in each, GEE post hoc Bonferroni’s test. ***P < 0.001. Boxes show the 25th–75th percentiles with the median value as a line within each box, and whiskers indicate the 10th and 90th percentiles of the data in **A**, **D**, and **E**. Data represent the mean ± SEM in **B** and **C**. All data points are shown as open circles

Macrophages differentiate into different phenotypes depending on various cues in the local tissue microenvironment. They are capable of polarizing toward a spectrum of phenotypes, including the classical (proinflammatory M1 phenotype) and alternative (anti-inflammatory M2 phenotype) activation states, regulatory phenotypes, and subtypes of these broad classifications [8]. In particular, M2 macrophages contribute primarily to axon regeneration [10, 39]. In line with the observation mentioned above, CD206, an M2 polarization marker, was overexpressed, whereas CD11c immunoreactivity was absent at the site of IAN injury 14 day post-IANX (Additional file 1: Fig. S7A, B). Western blot analysis also showed a significant increase in CD206 levels post-IANX, whereas CD11c was undetectable (CD206: Mann–Whitney *U* test, *P* < 0.0111; CD11c: Mann–Whitney *U* test, *P* = 0.9999; Additional file 1: Fig. S7C, D). To elucidate the necessity of macrophage-derived CTSS in sensory recovery post-IANX, we harvested rat peritoneal macrophages and polarized M2 macrophages through IL-4/IL-13-mediated stimulation and then performed an adoptive transfer of M2 macrophages at the injured site. During the cell separation procedure, IBA1-negative cells were eliminated (Additional file 1: Fig. S8A), indicating that the transplanted cells were macrophages. IL-4/IL-13-stimulated macrophages were positive for CD206, whereas lipopolysaccharide/interferon-γ-stimulated macrophages were not (Additional file 1: Fig. S8B). Hypoesthesia recovery post-IANX accelerated significantly after M2 macrophage transplantation compared with that of control medium administration (GEE post hoc Bonferroni’s test, *P* = 0.003, df = 1, Wald χ^2^ = 9.039; Fig. 3A), similar to that after rhCTSS administration (Fig. 2E). In contrast, the effects of M2 macrophage transplantation were inhibited by Z-FL treatment (GEE post hoc Bonferroni’s test, *P* = 0.002, df = 1, Wald χ^2^ = 9.794; Fig. 3A). Cultured macrophages showed significantly upregulated CTSS production and secretion upon IL-4/IL-13 stimulation (unpaired *t* test, *P* = 0.0008, t_(7)_ = 5.573; Fig. 3B, unpaired *t* test, *P* = 0.0025, t_(8)_ = 4.336; Fig. 3C). We further transplanted *Ctss*-knockdown M2 macrophages at the injured site 2 day post-IANX. *Ctss*-knockdown in M2 macrophages was confirmed using western blot analysis (Mann–Whitney *U* test, *P* = 0.0286; Fig. 3D). The adoptive transfer of *Ctss*-knockdown M2 macrophages significantly delayed recovery from hypoesthesia compared to transferring siCont-treated M2 macrophages (GEE post hoc Bonferroni’s test, *P* < 0.001, df = 1, Wald χ^2^ = 12.175; Fig. 3E). These results suggest that M2 macrophage-derived CTSS contributes to axon regeneration.

### CTSS processes Ephrin-B2 in rats and humans

**Fig. 4:**
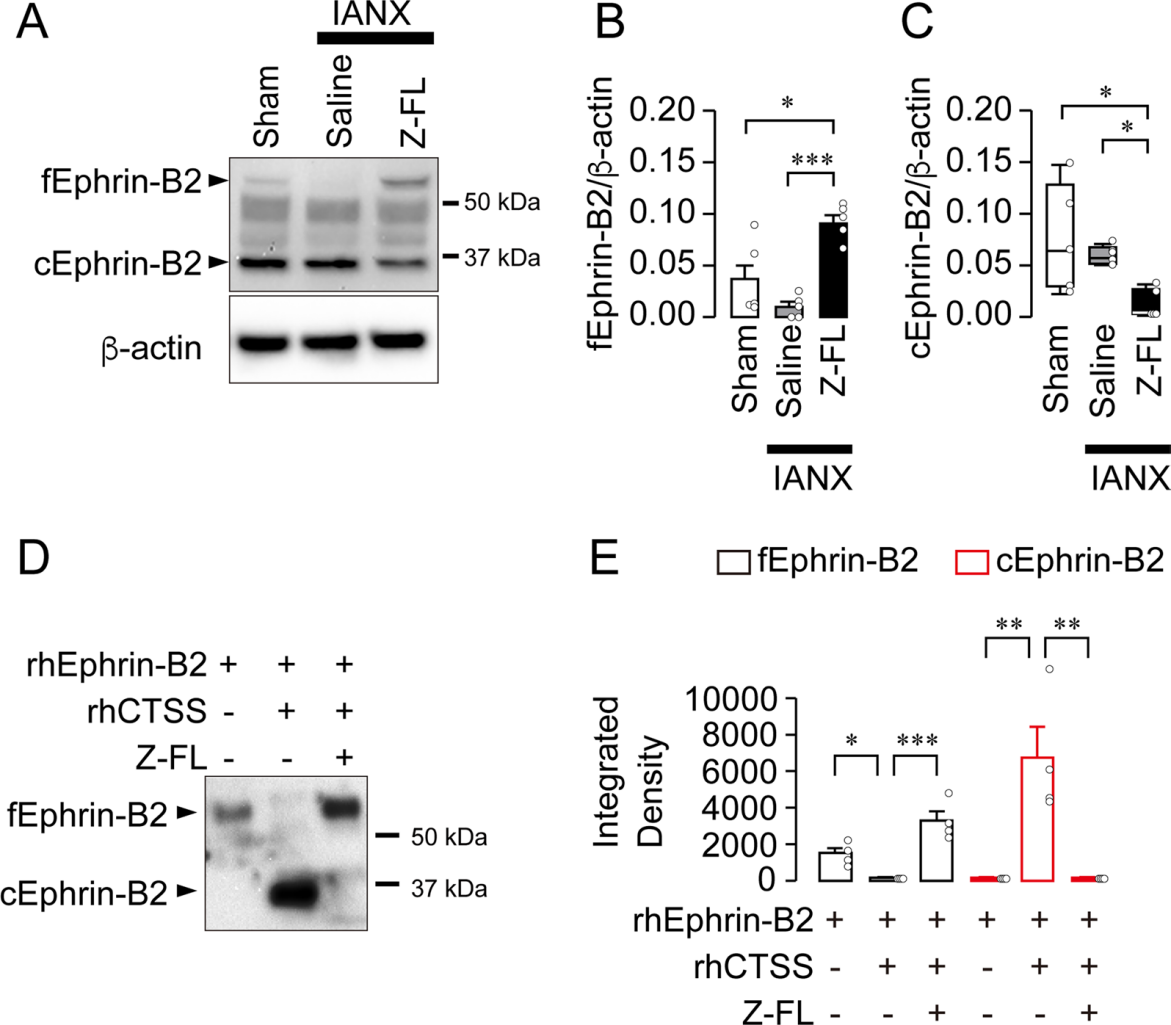
CTSS cleaves Ephrin-B2. **A** Blot of full-length Ephrin-B2 (fEphrin-B2) and cleaved Ephrin-B2 (cEphrin-B2) at the site of IAN injury at 14 day post-IANX. **B, C** Columns represent the average expression values of fEphrin-B2/β-actin (**B**) or cEphrin-B2/β-actin (**C**). *n* = 5 in each, one-way ANOVA post hoc Tukey’s test. *P < 0.05, ***P < 0.001 in **B**, Kruskal–Wallis post hoc Dunn’s test, *P < 0.05 in **C**. **D** Blot of fEphrin-B2 and cEphrin-B2 after mixing of recombinant human Ephrin-B2 (rhEphrin-B2) and recombinant human CTSS (rhCTSS). **E** Column represents the average values of the integrated density of fEphrin-B2 or cEphrin-B2. *n* = 4 independent experiments, one-way ANOVA post hoc Tukey’s test. *P < 0.05, **P < 0.01, ***P < 0.001. Data represent the mean ± SEM in **B**, **C**, and **E**. All data points are shown as open circles

CTSS is a lysosomal cysteine protease that exhibits proteolytic activity even in the extracellular space [29, 40, 41]. The trans-dimerization of Ephrin-B2–EphB2 signaling via cell-to-cell contact between fibroblasts and SCs is critically involved in axon regeneration after nerve injury [26]. Ephrin-B2 is a membrane-tethered ligand; however, it is cleaved into a shorter peptide form under certain circumstances [31]. It remains unclear whether the membrane-bound Ephrin-B2 contributes to axon regeneration after conversion to its shorter form. We thus speculated that CTSS-induced Ephrin-B2 cleavage occurred at the site of IAN injury during recovery from hypoesthesia. Full-length Ephrin-B2 (fEphrin-B2) was hard to detect at the injury site post-IANX, whereas the shorter form of Ephrin-B2 (cleaved Ephrin-B2 [cEphrin-B2]) was observed upon western blot analysis (Fig. 4A). Compared to saline-administered rats, the levels of fEphrin-B2 and cEphrin-B2 at the injured site increased and decreased, respectively, after Z-FL administration (fEphrin-B2: one-way ANOVA post hoc Tukey’s test, *P* = 0.0007, F_(2,12)_ = 13.94; cEphrin-B2: Kruskal–Wallis post hoc Dunn’s test, *P* = 0.0108; Fig. 4A–C). Ephrin-B2 cleavage occurred to a certain extent in sham rats (Fig. 4A). To confirm if CTSS directly acts on Ephrin-B2, we mixed rhCTSS and recombinant human Ephrin-B2 (rhEphrin-B2) in vitro. Ephrin-B2 was cleaved in the presence of CTSS, while Z-FL inhibited the Ephrin-B2 cleavage (one-way ANOVA post hoc Tukey’s test, fEphrin-B2: *P* = 0.0004, F_(2,9)_ = 20.56; cEphrin-B2: *P* = 0.0012, F_(2,9)_ = 15.7; Fig. 4D, E). Furthermore, immunohistochemical analysis showed that the expression of Ephrin-B2 and its receptor, EphB2, merged with αSMA, a fibroblast marker, and S100β, an SC marker, respectively (Additional file 1: Fig. S9). These results imply that cEphrin-B2 acts on EphB2 in SCs.

**Fig. 5:**
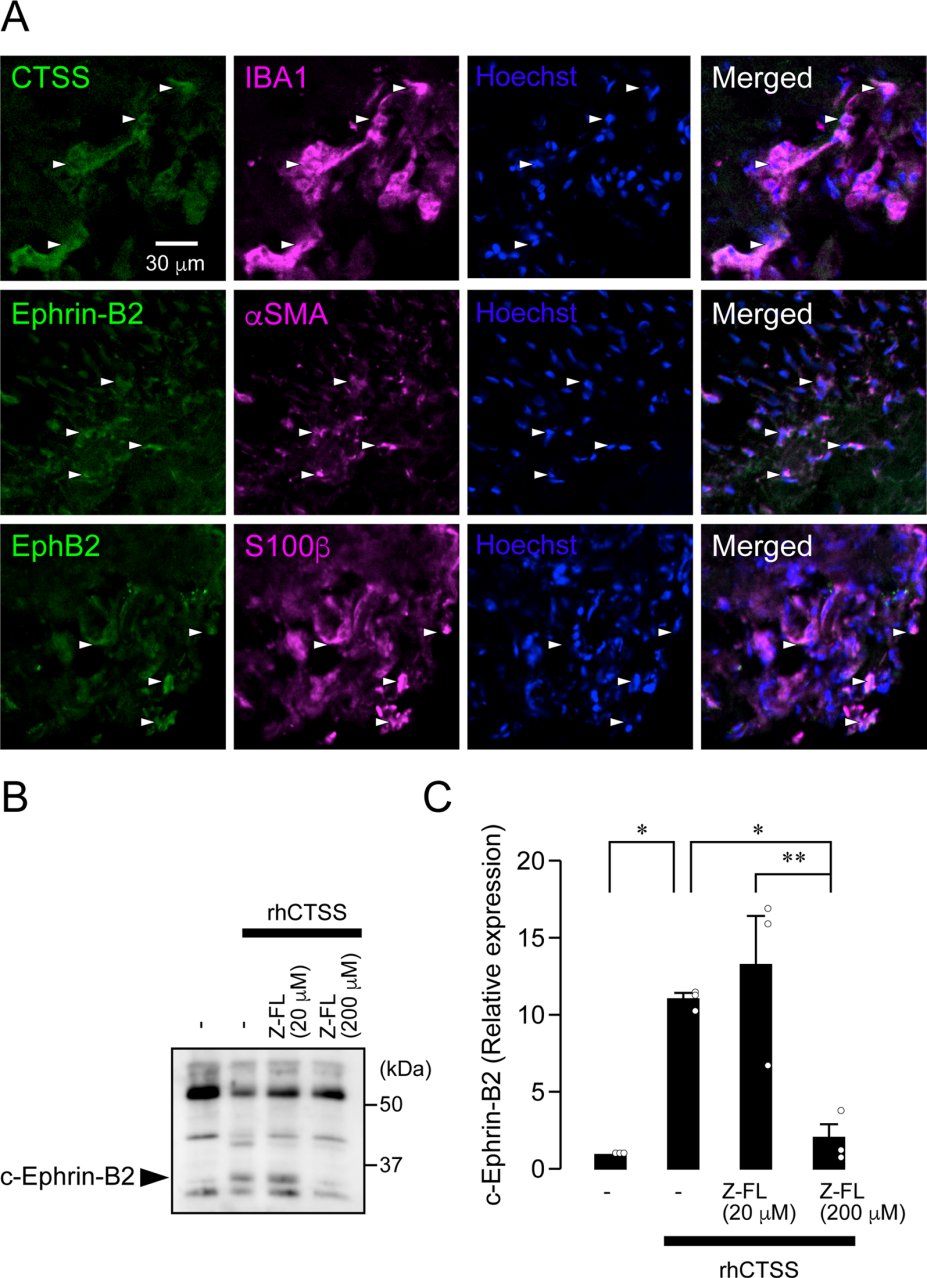
CTSS causes Ephrin-B2 cleavage in the human sensory nerve. **A** Images showing IBA1, CTSS, Ephrin-B2, αSMA, EphB2, or S100β immunofluorescence and Hoechst-33258 in the human nasopalatine nerve. Arrowheads indicate IBA1/CTSS-double-positive cells (top), Ephrin-B2/αSMA-double-positive cells (middle), and EphB2/S100β-double-positive cells (bottom). **B** Blots of fEphrin-B2 and cEphrin-B2 in the nasopalatine nerve homogenate after treatment with rhCTSS and Z-FL (20 or 200 μM). **C** Column represents the average values of fEphrin-B2/β-actin or cEphrin-B2/β-actin. *n* = 3 patients, one-way ANOVA post hoc Tukey’s test. *P < 0.05, **P < 0.01 All data points are shown in open circles

We further addressed whether CTSS-induced Ephrin-B2 cleavage is conserved in humans. The nasopalatine nerve, a sensory nerve, was excised from the incisive canals during surgery. We performed immunohistochemical analyses on the nasopalatine nerve and found that CTSS, Ephrin-B2, and EphB2 were expressed in macrophages, fibroblasts, and SCs, respectively (Fig. 5A). Next, we evaluated whether Ephrin-B2 in the homogenate of the nasopalatine nerve is cleaved by rhCTSS. The amount of cEphrin-B2 increased after rhCTSS treatment, which was inhibited in the presence of 200 μM Z-FL (one-way ANOVA post hoc Tukey’s test, *P* = 0.0016, F_(3, 8)_ = 0.8363; Fig. 5B, C). In contrast, 20 μM Z-FL did not inhibit rhCTSS-induced Ephrin-B2 cleavage (Fig. 5B, C).

### CTSS initiates fibroblast—SC signaling

**Fig. 6:**
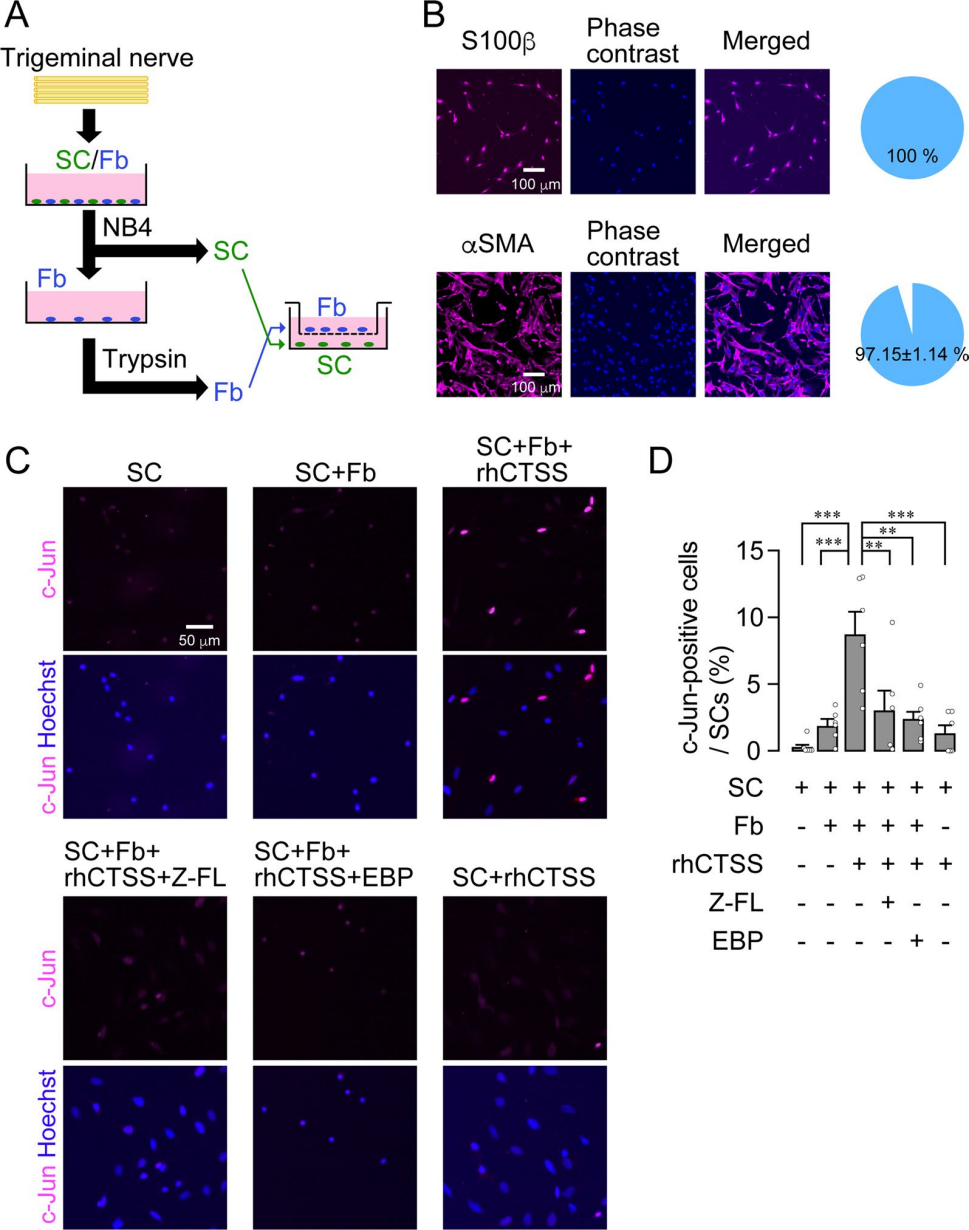
CTSS induces c-Jun in Schwann cells via the shedding of Ephrin-B2 on fibroblasts. **A** Schematic illustration of the isolation of Schwann cells (SCs) and fibroblasts (Fb) from the trigeminal nerve. **B** Images showing S100β-positive cells or αSMA-positive cells from isolated cells. Pie charts show the purity of S100β-positive cells or αSMA-positive cells isolated from mixed cultures of cells from the trigeminal nerve. *n* = 6 independent culture. **C** Images show c-Jun and Hoechst immunofluorescence in cultured SCs following treatment with rhCTSS, rhCTSS + Z-FL, or rhCTSS + Ephrin-B2 blocking peptide (EBP). **D** Column represents the average ratio of c-Jun-positive cells per SCs culture. *n* = 6 independent cultures, one-way ANOVA post hoc Tukey’s test. **P < 0.05, ***P < 0.01. All data points are shown in open circles

Cleaved Ephrin-B2 is expected to bind to SCs and convert them to a repair-supportive phenotype, leading to axon regeneration. Therefore, we made primary cultures of fibroblasts and SCs from the trigeminal nerves of neonatal rats. Each cell was isolated and cultured using cell culture inserts (upper: fibroblasts, lower: SCs) (Fig. 6A). The purity of the isolated SCs and fibroblasts was 100% and 97.15% ± 1.14%, respectively (Fig. 6B). The proportions of c-Jun-positive SCs were evaluated by immunohistochemical analysis after drug treatments, which were 0.28% ± 0.24% and 1.88% ± 0.49% in SC cultured alone and in co-culture of fibroblasts and SCs, respectively (Fig. 6C, D). rhCTSS treatment in fibroblast co-cultures significantly increased the number of c-Jun-positive SCs (8.69% ± 1.72%) compared with those in SCs cultured alone (one-way ANOVA post hoc Tukey’s test; Fig. 6C, D). The rhCTSS-induced increase in the number of c-Jun-positive SCs upon fibroblast co-culture was markedly suppressed by treatment with Z-FL (3.01% ± 1.51%, one-way ANOVA post hoc Tukey’s test; Fig. 6C, D) and EBP treatments (2.35% ± 0.63%, one-way ANOVA post hoc Tukey’s test; Fig. 6C, D). rhCTSS-induced c-Jun induction was not observed in SCs cultured alone (1.29% ± 0.59%, Fig. 6C, D). These results indicate that CTSS-cleaved Ephrin-B2 converts SCs into a repair-supportive phenotype.

To examine whether CTSS directly affects axons during regeneration, we cultured primary TG neurons and separated them into axonal and cell body compartments using a microfluidics culture system (Additional file 1: Fig. S10A). After 7 days of culture, we treated the axon compartment with rhCTSS or saline and analyzed the axon lengths. There was no significant difference in axon lengths between the rhCTSS and saline treatment groups (Mann–Whitney *U* test, *P* = 0.1534; Additional file 1: Fig. S10B, C), suggesting that CTSS does not directly affect axons.

### CTSS-induced Ephrin-B2 liberation promotes axonal regeneration

**Fig. 7:**
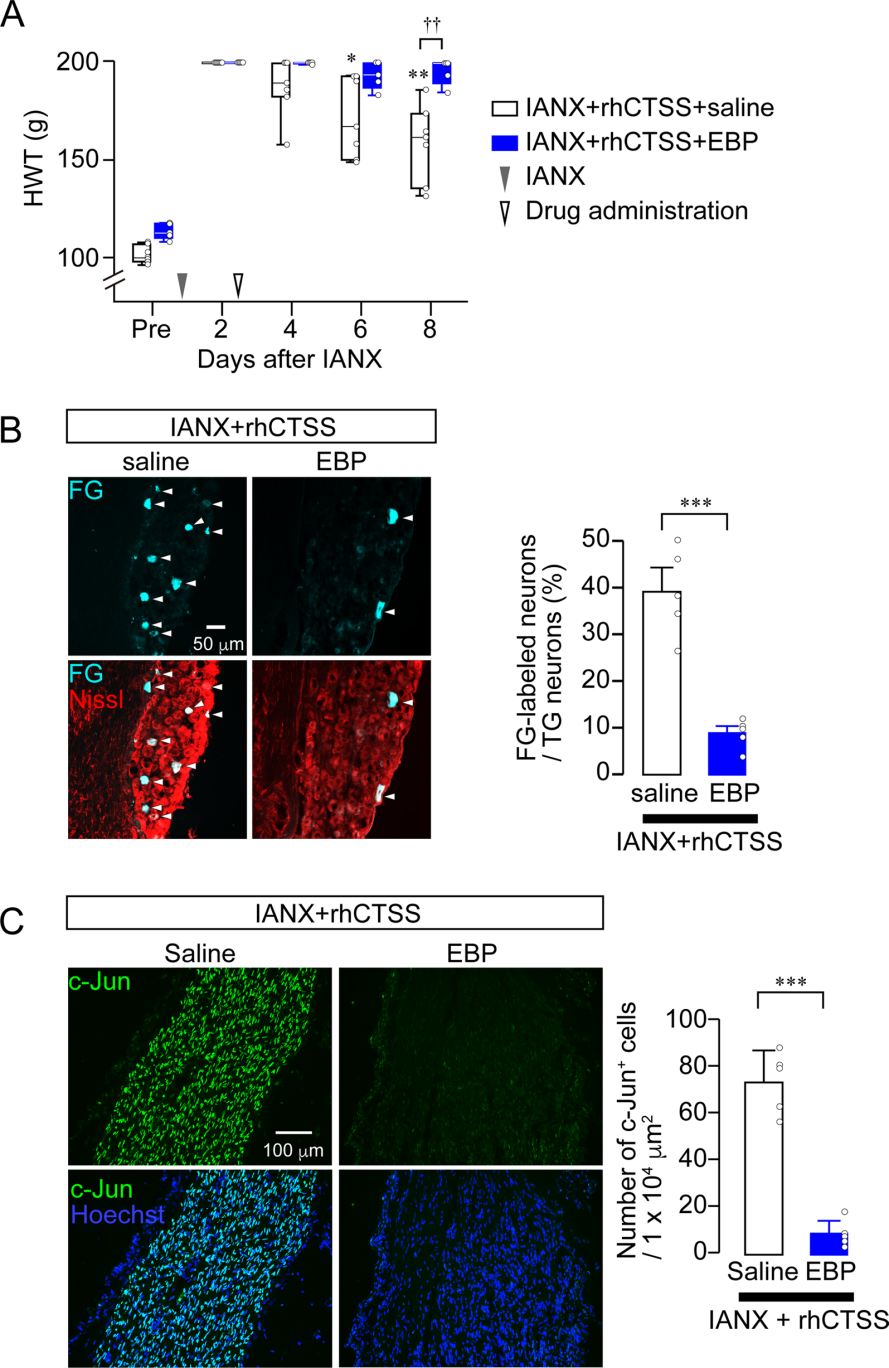
CTSS-induced Ephrin-B2 liberation accelerates sensory recovery. **A** Time course of HWT in IANX rats treated with rhCTSS + saline or rhCTSS + EBP. IANX + rhCTSS + saline (*n* = 7); IANX + rhCTSS + EBP (*n* = 5), Friedman test post hoc Dunn’s test, *P < 0.05, **P < 0.01 vs. day 2; GEE post hoc Bonferroni’s test. ^††^*P* < 0.01. Boxes show the 25th–75th percentiles with the median value as a line within each box, and whiskers indicate the 10th and 90th percentiles of the data. All data points are shown in open circles. **B** Images showing FG-labeled TG neurons 14 day post-IANX. Column represents the average number of FG-labeled TG neurons out of total TG neurons. *n* = 5 in each, unpaired *t* test. ***P < 0.001. **C** Images show c-Jun and Hoechst-33258 immunofluorescence at the site of IAN injury 14 day post-IANX. Column represents the average number of c-Jun-positive cells at the injured site. *n* = 5 in each, unpaired *t* test. ***P < 0.001. Data represent the mean ± SEM in **B** and **C**. All data points are shown as open circles

To examine whether CTSS-induced Ephrin-B2 liberation contributes to axon regeneration in vivo, we administered rhCTSS + saline or rhCTSS + EBP to the site of IAN injury 2 day post-IANX. A significant sensory recovery in rhCTSS + saline-administered rats was observed at 6 day post-IANX (Friedman test post hoc Dunn’s multiple comparison test, *P* = 0.0033, vs. day 2; Fig. 7A). Conversely, the rhCTSS-induced improvement in sensory recovery was significantly suppressed in the presence of EBP (Friedman test post hoc Dunn’s multiple comparison test, *P* = 0.1042, vs. day 2; Fig. 7A). Statistical difference between the groups was observed at 6 days after drug administration (GEE post hoc Bonferroni’s test, *P* < 0.001, df = 1, Wald χ^2^ = 27.752; Fig. 7A). Aside from the recovery from hypoesthesia, the connection between the proximal and distal stumps of the IAN, as identified by the existence of FG-labeled TG neurons in the TG, was investigated at 8 day post-IANX. Administering EBP significantly reduced the number of FG-labeled TG neurons (IANX + rhCTSS + saline: 38.96% ± 4.18%; IANX + rhCTSS + EBP: 8.83% ± 1.35%, unpaired *t* test, *P* = 0.0001, t_(7)_ = 6.858; Fig. 7B).

We further addressed whether c-Jun expression at the site of IAN injury was influenced by EBP administration. The number of c-Jun-positive cells at the injury site in rhCTSS + EBP-administered rats post-IANX was significantly lower than in rhCTSS + saline-administered rats post-IANX (IANX + rhCTSS + saline: 73.79 ± 6.07 cells/1 × 10^4^ μm^2^; IANX + rhCTSS + EBP: 8.85 ± 2.53 cells/1 × 10^4^ μm^2^; unpaired *t* test, *P* < 0.0001, t_(8)_ = 9.869; Fig. 7C). In contrast, the number of IBA1-positive cells at the injured site remained unchanged after EBP administration (unpaired *t* test, *P* = 0.248, t_(8)_ = 1.246; Additional file 1: Fig. S11), suggesting that EBP did not reduce the number of macrophages.

## Discussion

**Fig. 8:**
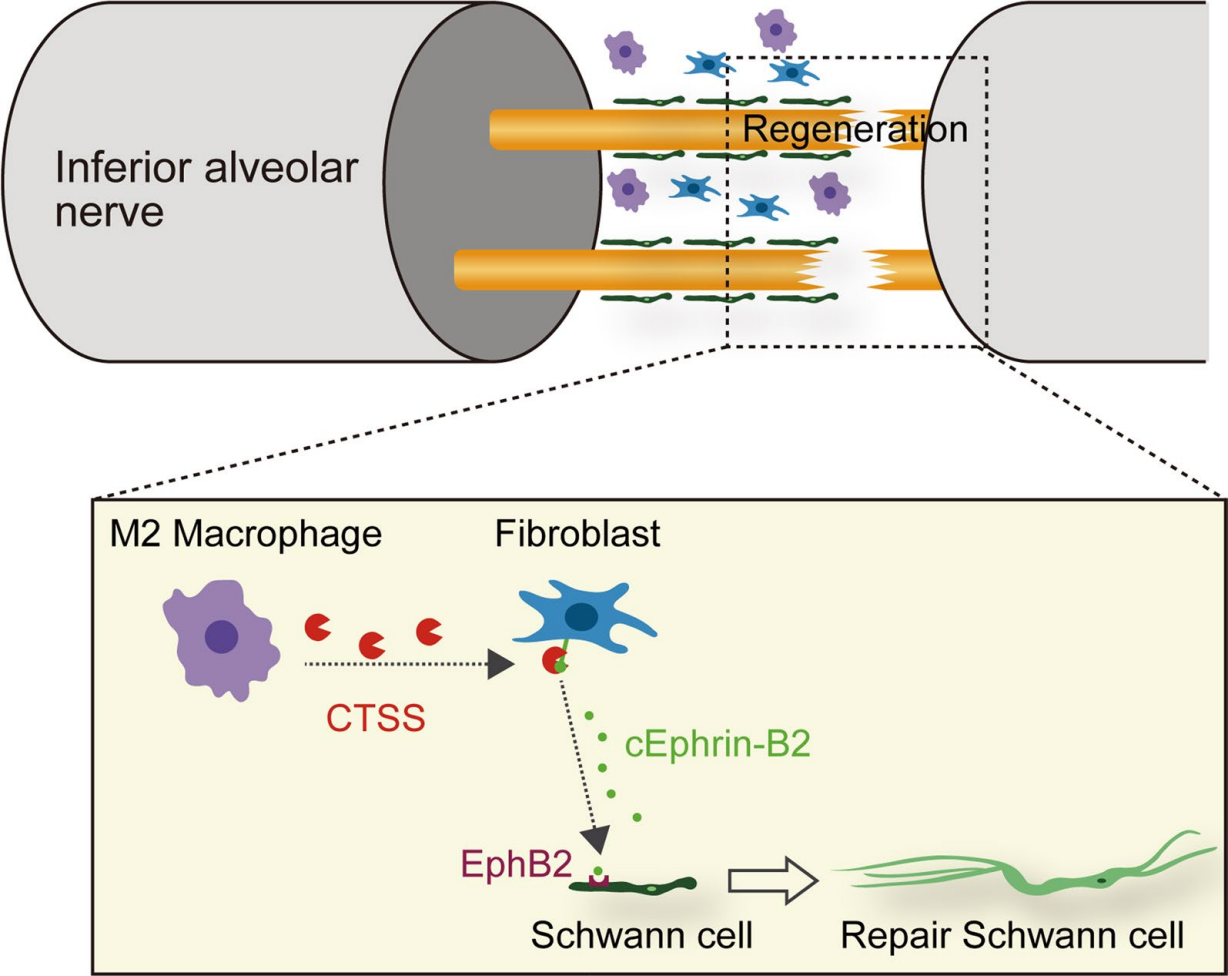
Schematic illustration of the proposed model of axon regeneration at the site of IAN injury. M2 macrophages accumulate at the injured site post-IANX. M2 macrophages then release CTSS, which liberates Ephrin-B2 on fibroblasts. Ephrin-B2 then binds to EphB2 on SCs, inducing SCs to change into repair phenotype, subsequently facilitating axon regeneration

Intercellular signaling between macrophages and SCs has recently been implicated in axon regeneration after peripheral nerve injury [23]. However, the role of axon regeneration factors in intercellular communication remains unclear. Combining transcriptome and database analyses, we found that CTSS was upregulated in the IAN post-IANX and was exclusively expressed in macrophages, contributing to axon regeneration and sensory recovery. Pharmacological inhibition or genetic silencing of CTSS at the site of IAN injury significantly delayed axon regeneration, whereas rhCTSS supplementation at the injury site accelerated axon regeneration. Adoptive transfer of M2 macrophages at the injury site accelerated axon regeneration, and this effect was abrogated by *Ctss* knockdown in M2 macrophages. CTSS liberated Ephrin-B2 from fibroblasts, which induced robust c-Jun expression in SCs via EphB2. The CTSS-mediated promotion of axon regeneration and c-Jun expression at the injured site was suppressed by Ephrin-B2 signaling. CTSS-induced Ephrin-B2 cleavage was observed in the sensory nerve in human patients. These results indicate that macrophage-derived CTSS initiates Ephrin-B2 shedding, which triggers the transformation of SCs into a repair-supportive phenotype, activating the intrinsic regenerative capacity of axons after peripheral nerve injury (Fig. 8).

Multiple studies reported that macrophage dysfunction markedly delayed axon regeneration in animal models, as described below. Macrophages eliminate the debris at the injured site, in this case, creating a regenerative environment around the injured axon [6]. In addition, macrophages are involved in axon regeneration by secreting multiple substances. Slit3, a repulsive factor, tightly controls the direction of axon extension by inhibiting axon outgrowth in undesired orientations [42]. Conversely, uPA promotes axon outgrowth by acting on the growth cone [39]. Other molecules, such as IL-6, Gas6, and Hbegf, enable SCs to acquire repair-supportive phenotypes [23]. Macrophages also recruit the blood vessels necessary for SC scaffolding and promote angiogenesis between the proximal and distal stumps via VEGF-A, facilitating axon regeneration [22]. Accordingly, macrophages play essential roles in initiating and maintaining axon regeneration. In this study, we found that CTSS, a macrophage-derived lysosomal protease, is required for axon regeneration.

As described above, multiple macrophage-secreted factors are involved in axon regeneration. However, unlike in the sciatic nerve [23, 42], *Gas6*, *Hbegf*, or *Slit3* mRNA levels remained unchanged in the IAN after nerve injury (Additional file 2). It remains unclear why distinct gene profiles were observed between tissues. Differential gene expression in macrophages accumulated at the IAN and sciatic nerve may be attributed to the tissue environment surrounding these nerves. Ydens et al. reported differential gene expression profiles in resident macrophages in the optic and sciatic nerves [43], indicating that regional heterogeneity determines the differences in gene expression patterns. The characteristic difference between the IAN and sciatic nerves is their surrounding environment, i.e., IAN passes through the mandible bone and runs parallel to the artery in the mandibular canal [44]. Studies have shown that bone- and artery-derived molecules cause axon outgrowth and remyelination, respectively [45, 46]. The above factors probably alter the gene profiles of resident and accumulated macrophages in the IAN, unlike in the sciatic nerve and other areas. Notably, while macrophages exhibit regional heterogeneity, the expression of *Ctss* mRNA is common among resident macrophages in several regions [47]. Moreover, our western blots detected CTSS protein in the intact IAN. Considering the prevalent expression of CTSS in the peripheral nerves, the regenerative effect of CTSS may occur in both sensory and motor nerves. Therefore, CTSS is presumably effective in recovery from paralysis due to motor nerve injury.

Despite being a lysosomal protease, CTSS is secreted in the extracellular space and could cause extracellular proteolysis under neutral pH in the central nervous system, liberating fractalkine [29] and degrading the extracellular matrix [30]. In this study, we identified that Ephrin-B2 is a novel molecular target of CTSS. The role of membrane-tethered Ephrin-B2 in fibroblasts has been implicated in axon regeneration [26]. Parrinello et al. reported that physical contact between fibroblasts and SCs via Ephrin-B2 is essential for axon regeneration, as phenotypic changes occurred in SCs when co-cultured with fibroblasts but not when co-cultured using cell culture inserts [26]. In contrast, based on our results, the CTSS-mediated shedding of Ephrin-B2 from fibroblasts is involved in axon regeneration. Several reasons could explain these different results. Ephrin-B2 is a membrane-tethered ligand, and the cross-talk between fibroblasts and SCs is believed to occur via the trans-dimerization of Ephrin-B2-EphB2 upon cell-to-cell contact [26]. In this study, we could not detect c-Jun expression in SCs when SCs and fibroblasts were co-cultured using cell culture inserts, in agreement with Parrinello’s findings. The rhCTSS-mediated stimulation of co-cultured SCs and fibroblasts induced c-Jun expression in SCs, which neutralized by Ephrin-B2. We could not observe c-Jun expression in CTSS-treated pure SC cultures. Considering these findings, intercellular signaling between fibroblasts and SCs via direct cell-to-cell contact and soluble factors synergistically facilitates phenotypic change in SCs during axon regeneration.

In the present study, CTSS did not directly influence the axon outgrowth of TG neurons and c-Jun induction in SCs. However, we could not exclude the possibility that CTSS is involved in biological activities other than Ephrin-B2 liberation. CTSS facilitates angiogenesis in cancerous tissue [48], and newly generated blood vessels between the distal and proximal stumps are essential for axon regeneration, as described above. SCs align alongside blood vessels, facilitating axon regeneration [22]. Accordingly, it is plausible that CTSS promotes angiogenesis at the site of IAN injury. Another possible role of CTSS could be in fibroblast activation. Protease-activated receptor (PAR) has membrane-tethered ligands within its extracellular domain, and CTSS acts as a biased agonist of PAR [49]. Fibroblasts express PAR2, and PAR2 activation causes fibroblast proliferation [50]. Therefore, CTSS possibly causes sufficient fibroblast proliferation to serve as a source of Ephrin-B2 during axon regeneration. Indeed, *Efnb2* mRNA was significantly upregulated post-IANX based on our transcriptome data. In future studies, we need to elucidate other roles of CTSS in axon regeneration.

The importance of macrophages in axon regeneration has been reported [13–15]. Specifically, M1-type macrophages are transiently observed at the injured site of peripheral nerves in the early phase of injury, which shift toward the M2 phenotype when axon regeneration occurs [10]. Upon sensory recovery, we detected CD206^+^ but not CD11c^+^ macrophages at the injured IAN. Despite an incomplete recovery, the accumulated macrophages egress from the injury site [51, 52]. The loss of regeneration-promoting signals from macrophages might be a cause of the insufficient regenerative capacity of peripheral nerves after their injury. Notably, c-Jun expression in SCs is not sustained throughout axon regeneration in the sciatic nerve. It is dramatically reduced 10 weeks after injury, whereas c-Jun overexpression in SCs supported long-lasting axon regeneration [24]. We found that macrophages are critically involved in c-Jun induction in SCs at the injured IAN. Hence, it is likely that long-term contact between macrophages and SCs is essential for the sustained regeneration of axons after their injury. As abundant fibroblasts are located at the injured nerve [26], CTSS treatment at the injury site may induce effective and long-lasting regenerative programs without intercellular signaling between macrophages and SCs.

Several studies reported that the pharmacological inhibition or gene ablation of CTSS ameliorated or suppressed inflammatory diseases [29, 53]. To the best of our knowledge, the therapeutic effect of CTSS has not been reported. However, it has been suggested that macrophages that accumulate during tissue repair express CTSS [54]. In addition, M2-polarized macrophages exhibit an increased synthesis of CTSS [55], consistent with our results. Hence, CTSS expressed in repair-supportive macrophages has potent therapeutic effects on axon regeneration.

We can then propose a novel therapeutic strategy for nerve damage based on CTSS. The direct treatment of CTSS to damaged nerves during tissue reconstruction, such as when nerves are damaged in an accident or injury, may promote the recovery of motor and sensory functions. This treatment might overcome several problems during axon regeneration. In surgeries to reconstruct the nerves, the severed nerve segments are stitched together, or nerve grafts are used to fill the gap between the distal and proximal stumps [56]. The degree of functional recovery depends on the gap distance between the distal and proximal stumps [56]; however, tissue function is not always fully restored. In our study, CTSS promoted the reconnection of the severed IAN. Thus, we believe that CTSS is a good therapeutic candidate for nerve damage in humans. Amputation neuromas are painful nodular lesions that occur at the proximal end of amputation and are a potential problem when a peripheral nerve is damaged or injured [57]. When the ends of a severed nerve are too far apart or one end is missing, the regenerating nerve can lose direction and cause an abundance of local nerve growth factors and SC proliferation. This results in the formation of scar tissue and an increase in collagen accumulation, ultimately leading to the development of neuromas [58, 59]. This is presumably due to the loss of Ephrin-B2, which can organize the direction of axon growth [26]. It is likely that CTSS treatment can inhibit the formation of such neuromas by orienting the direction of the nerve and causing sustained regeneration.

Protein therapeutics are attracting worldwide attention due to their advantages, such as the specificity of molecular targets and low immune response, being biological substances [60, 61]. In the present study, recombinant CTSS promoted axon regeneration; hence, our study provided data on the potential advantage of CTSS as a beneficial therapy for nerve damage. We believe that administering a sustained-release formulation of CTSS to damaged nerves can effectively improve sensory and motor functions in patients. In addition, treatment with fewer side effects can be achieved by the local administration of CTSS.

Our study has certain limitations. We used human sensory nerves; they are acutely excised during surgery but not from the damaged nerves during the repair process. Currently, it is ethically challenging to use damaged human nerves for experiments. The nasopalatine nerve, which is part of the second branch of the trigeminal nerves, differs from the IAN, the third branch of the trigeminal nerve. It is impossible to utilize the human IAN for our experiments. Nevertheless, we found that CTSS-induced Ephrin-B2 liberation and molecular expression profiles, including those of CTSS, Ephrin-B2, and EphB2, in the IAN of rats, are conserved in human sensory nerves. Although it remains unclear whether CTSS has therapeutic effects on motor nerves and axons in the central nervous system after their damage, our study offers new insights into axon regeneration from this perspective.

Our findings demonstrate that M2 macrophage-derived CTSS plays a critical role in axon regeneration. Our study highlights the importance of the macrophage–fibroblast–SC-axon quadripartite signal relay in axon regeneration and is expected to provide new biological clues for elucidating the mechanisms underlying nerve injury repair. Furthermore, understanding how cell-to-cell communication regulates axonal regeneration will open new avenues for effective therapeutic intervention after peripheral nerve injury.

### Supplementary Information

**Additional file 1: Figure S1. **Schematic drawings of each experiment. **Figure S2 **Axon regeneration of IAN post-IANX. A Photographs show the site of IAN injury 14 day post-IANX. Broken lines indicate the edge of IAN. Arrows indicate the injured site. B Immunofluorescent images show NF200 and Hoechst at the injured site post-IANX. Arrows indicate the injured site. **Figure S3. **c-Jun expression at the site of IAN injury 14 day post-IANX. Images show c-Jun, IBA1, or αSMA immunofluorescence at the injured site. Pie charts indicate c-Jun-positive cells per IBA1-positive cells or c-Jun-positive cells per αSMA-positive cells. n = 5. **Figure S4.** Differentially expressed genes in the IAN after its injury. Hierarchical clustering of three IANs from sham and IANX rats based on differentially expressed RNA transcripts. Each column represents a sample and each row represents a transcript. The expression level of each gene in a single sample is depicted according to the color scale. **Figure S5.** Metascape bar graph for viewing top enrichment clusters. Macrophage-selective genes were picked up from IAN DEGs data using the Harmonizome database, and these were bar-plotted using Metascape. The length of each bar represents –log_10_ (*p *value). The left shows GO terms. **Figure S6. **Knockdown efficacy of CTSS after siRNA administration at the site of IAN injury post-IANX. Blot of CTSS at the injured site 14 day post-IANX. siCont and siCtss indicate negative control siRNA and Ctss siRNA, respectively. The column represents the average values of CTSS/β-actin. n = 5 in each, unpaired *t* test, *P < 0.05. Data represent the mean ± SEM. All data points are shown in open circles. **Figure S7. **Macrophage phenotype at the site of IAN injury. A, B Images show CD206, CD11c, or IBA1 at the injured site 14 day post-IANX. Arrowheads indicate CD206 and IBA1 double-positive cells. C, D Blots show CD206 (C) and CD11c (D) at the injured site 14 day post-surgery. The column represents CD206/β-actin (C) or CD11c/β-actin (D). CD11c expression was undetectable at the injured site. n = 8 in each, Mann–Whitney *U* test, *P < 0.05. Boxes show the 25th–75th percentiles with the median value as a line within each box, and whiskers indicate the 10th and 90th percentiles of the data in (C). All data points are shown in open circles. **Figure S8. **Polarization of peritoneal macrophages. A Peritoneal macrophages were seeded on the plate and then non-adhesive cells were washed out. The images show IBA1 and Hoechst of the adhesive cells in the culture plate. The pie chart indicates IBA-positive cells per Hoechst-positive cells. n = 10 independent culture. B Macrophages were stimulated with IL-4 and IL-13 or LPS and IFNγ. The images show CD206 and differential interference contrast of macrophages. **Figure S9. **Expression of Ephrin-B2 or EphB2 at the site of IAN injury 14 day post-IANX. Images show Ephrin-B2, αSMA, EphB2, or S100β immunofluorescence at the injured site. Arrowheads indicate Ephrin-B2 and αSMA double-positive cells or EphB2 and S100β double-positive cells. **Figure S10. **Effects of rhCTSS on axon outgrowth in primary trigeminal ganglion (TG) neurons. A Schematic illustration of a chamber of compartmentalized neuronal cultures and time course of the preparation. B Images show β3-Tublin immunofluorescence in primary TG neurons. The column represents the average length of axons after treatment with saline or rhCTSS. n = 13 independent cultures, Mann–Whitney *U* test, *P *= 0.1534. Boxes show the 25th–75th percentiles with the median value as a line within each box, and whiskers indicate the 10th and 90th percentiles of the data. All data points are shown in open circles. **Figure S11. **Effects of Ephrin-B2 blocking peptide (EBP) on macrophage expression at the site of IAN injury 8 day post-IANX. Images show IBA1-positive cells at the injured site. The column represents the average values of the area occupied by IBA1 immunofluorescence at the injured site. n = 5 in each, unpaired *t* test, *P *= 0.248. All data points are shown in open circles. **Figure S12 **Original blot for Figure 2D (A), Figure 3B (B), and Figure 3C (C). The area enclosed by the red broken line is the image used in the figure. **Figure S13 **Original blot for Figure 4A (A), Figure 4D (B), and Figure 5B (C). The area enclosed by the red broken line is the image used in the figure. **Figure S14 **Original blot for Figure S6 (A), Figure S7C (B), and Figure S7D (C). The area enclosed by the red broken line is the image used in the figure.

**Additional file 2: Table S1.** Differential expression genes in IAN in IANX and sham rats 14 days after IANX.

**Additional file 3: Table S2.** Macrophage selective expressing genes analysis.

## Data Availability

The authors confirm that the data supporting the findings of this study are available within the article and its Supplementary material. The data sets used and/or analyzed during the present study are available from the corresponding author upon reasonable request. RNA-seq data have been deposited in NCBI GEO under accession number GSE232487.

## Abbreviations

SC: Schwann cell
CTSS: Cathepsin S
IAN: Inferior alveolar nerve
IANX: Inferior alveolar nerve transection
STAT3: Signal transducer and activator of transcription 3
IL: Interleukin
Gas6: Growth-arrest-specific gene 6
Clo: Clophosome-A
Lipo: Control liposome
rhCTSS: Recombinant human cathepsin S
EBP: Ephrin-B2 blocking peptide
Z-FL: Z-FL-COCHO
siRNA: Small interference RNA
siCtss: Ctss siRNA
siCont: Control siRNA
PFA: Paraformaldehyde
FG: Fluoro-Gold
PBS: Phosphate-buffered saline
DMEM: Dulbecco’s Modified Eagle Medium
FBS: Fetal bovine serum
HBSS: Hank’s Balanced Salt Solution
MEM: Eagle’s Minimum Essential Medium
HWT: Head withdrawal threshold
NF200: Neurofilament 200
TG: Trigeminal ganglion
RNA-seq: RNA-sequencing
fEphrin-B2: Full-length Ephrin-B2
cEphrin-B2: Cleaved Ephrin-B2
PAR: Protease-activated receptor
