# Supplementary material for: M2 macrophage-derived cathepsin S promotes peripheral nerve regeneration via fibroblast–Schwann cell-signaling relay
Source: J Neuroinflammation. 2023 Nov 9;20:258. doi: 10.1186/s12974-023-02943-2 (PMC10636844; doi:10.1186/s12974-023-02943-2)
Supplement: Supplementary file 1 — Additional file 1: Figure S1. Schematic drawings of each experiment. Figure S2 Axon regeneration of IAN post-IANX. A Photographs show the site of IAN injury 14 day post-IANX. Broken lines indicate the edge of IAN. Arrows indicate the injured site. B Immunofluorescent images show NF200 and Hoechst at the injured site post-IANX. Arrows indicate the injured site. Figure S3. c-Jun expression at the site of IAN injury 14 day post-IANX. Images show c-Jun, IBA1, or αSMA immunofluorescence at the injured site. Pie charts indicate c-Jun-positive cells per IBA1-positive cells or c-Jun-positive cells per αSMA-positive cells. n = 5. Figure S4. Differentially expressed genes in the IAN after its injury. Hierarchical clustering of three IANs from sham and IANX rats based on differentially expressed RNA transcripts. Each column represents a sample and each row represents a transcript. The expression level of each gene in a single sample is depicted according to the color scale. Figure S5. Metascape bar graph for viewing top enrichment clusters. Macrophage-selective genes were picked up from IAN DEGs data using the Harmonizome database, and these were bar-plotted using Metascape. The length of each bar represents –log10 (p value). The left shows GO terms. Figure S6. Knockdown efficacy of CTSS after siRNA administration at the site of IAN injury post-IANX. Blot of CTSS at the injured site 14 day post-IANX. siCont and siCtss indicate negative control siRNA and Ctss siRNA, respectively. The column represents the average values of CTSS/β-actin. n = 5 in each, unpaired t test, *P < 0.05. Data represent the mean ± SEM. All data points are shown in open circles. Figure S7. Macrophage phenotype at the site of IAN injury. A, B Images show CD206, CD11c, or IBA1 at the injured site 14 day post-IANX. Arrowheads indicate CD206 and IBA1 double-positive cells. C, D Blots show CD206 (C) and CD11c (D) at the injured site 14 day post-surgery. The column represents CD206/β-actin (C) or CD11c/β-actin [file 12974_2023_2943_MOESM1_ESM.docx]

**Additional file for**

**M2 Macrophage-derived cathepsin S promotes peripheral nerve regeneration via fibroblast–Schwann cell signaling relay**

Eri Oshima *et al*.

Corresponding author: Yoshinori Hayashi, hayashi.yoshinori@nihon-u.ac.jp

**The file includes:**

Supplementary figure and legends: figure S1 to S14

**
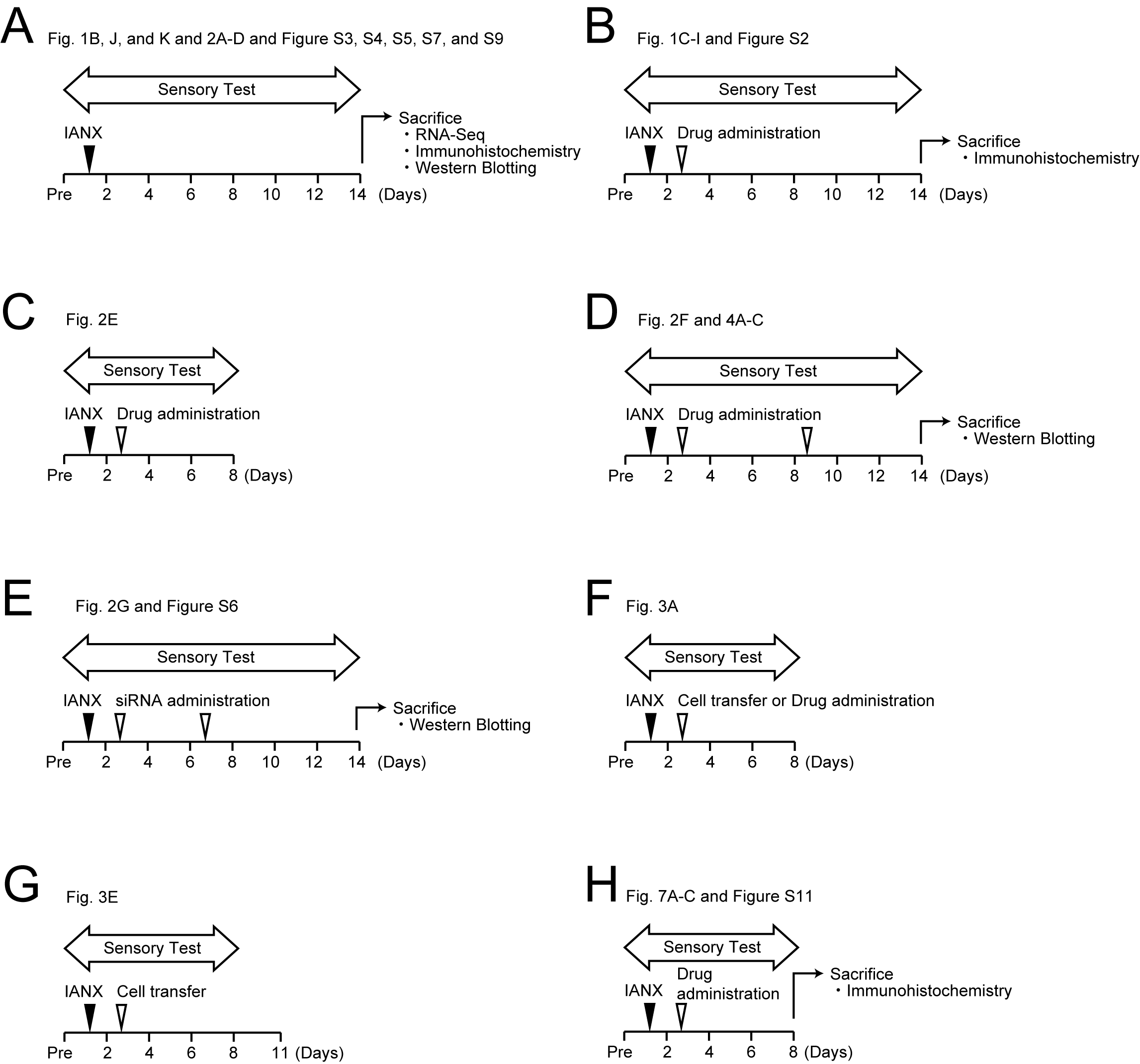
**

**Figure S1 Schematic drawings of each experiment.**


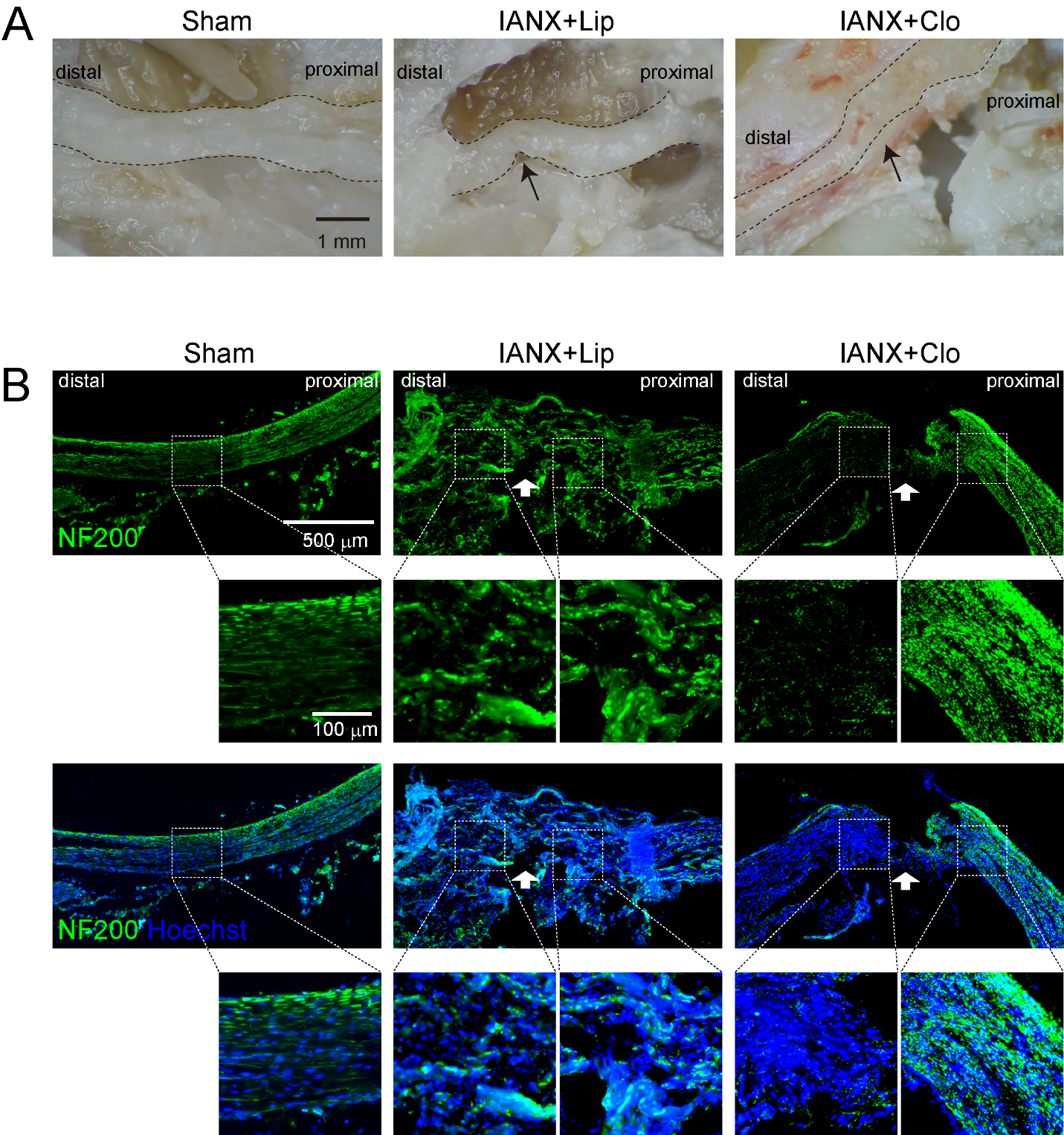


**Figure S2 Axon regeneration of IAN post-IANX. A** Photographs show the site of IAN injury 14 days post-IANX. Broken lines indicate the edge of IAN. Arrows indicate the injured site. **B** Immunofluorescent images show NF200 and Hoechst at the injured site post-IANX. Arrows indicate the injured site.


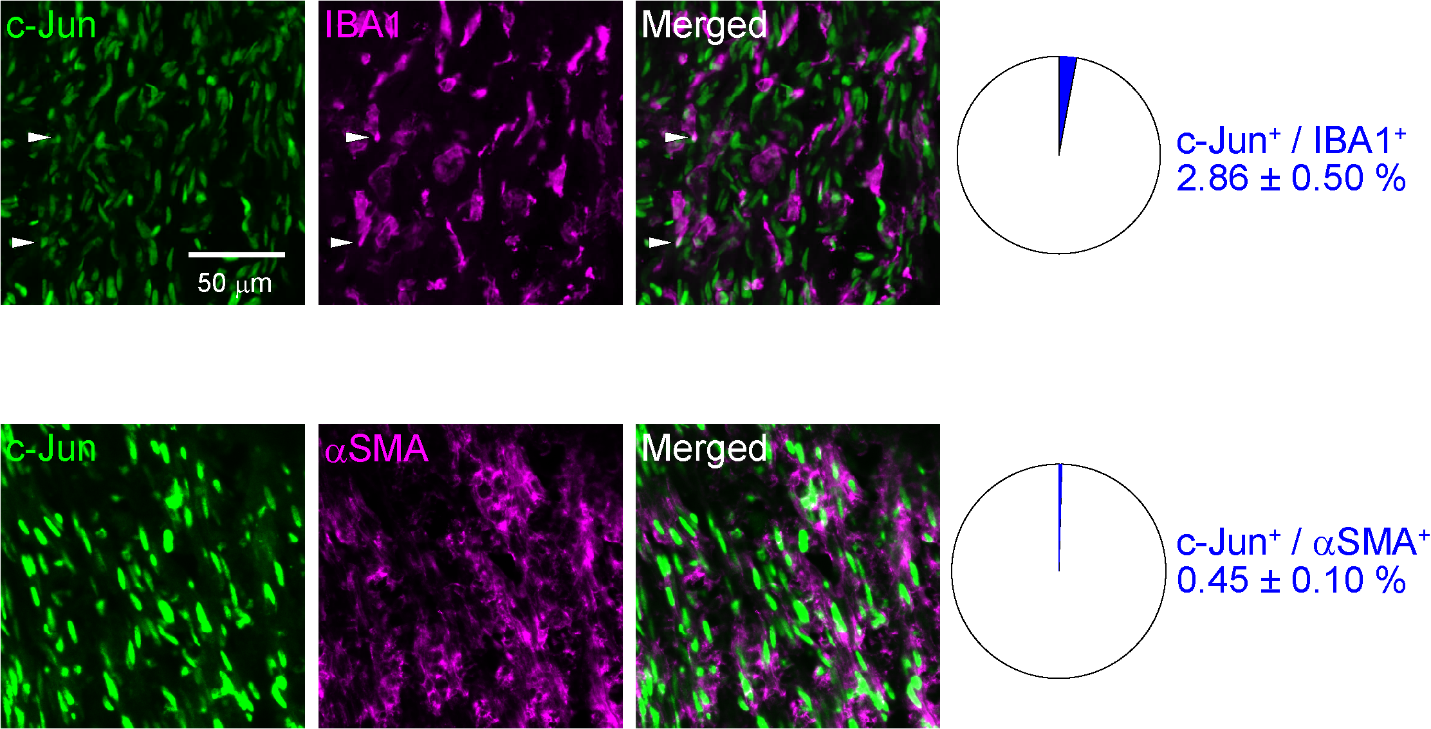


**Figure S3. c-Jun expression at the site of IAN injury 14 days post-IANX.** Images show c-Jun, IBA1, or αSMA immunofluorescence at the injured site. Pie charts indicate c-Jun-positive cells per IBA1-positive cells or c-Jun-positive cells per αSMA-positive cells. n = 5.

**
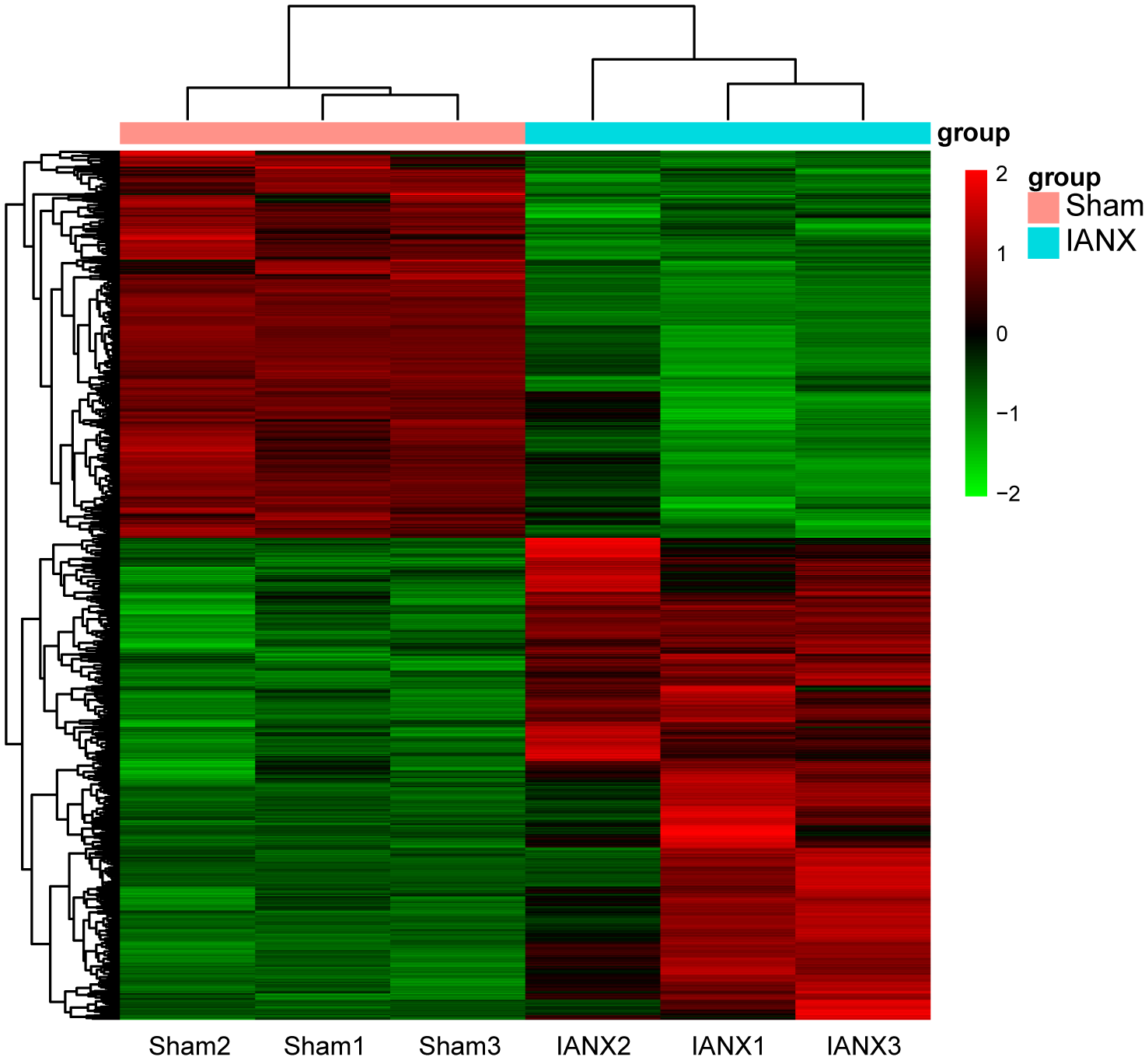
 Figure S4.** Differentially expressed genes in the IAN after its injury. Hierarchical clustering of three IANs from sham and IANX rats based on differentially expressed RNA transcripts. Each column represents a sample and each row represents a transcript. The expression level of each gene in a single sample is depicted according to the color scale.


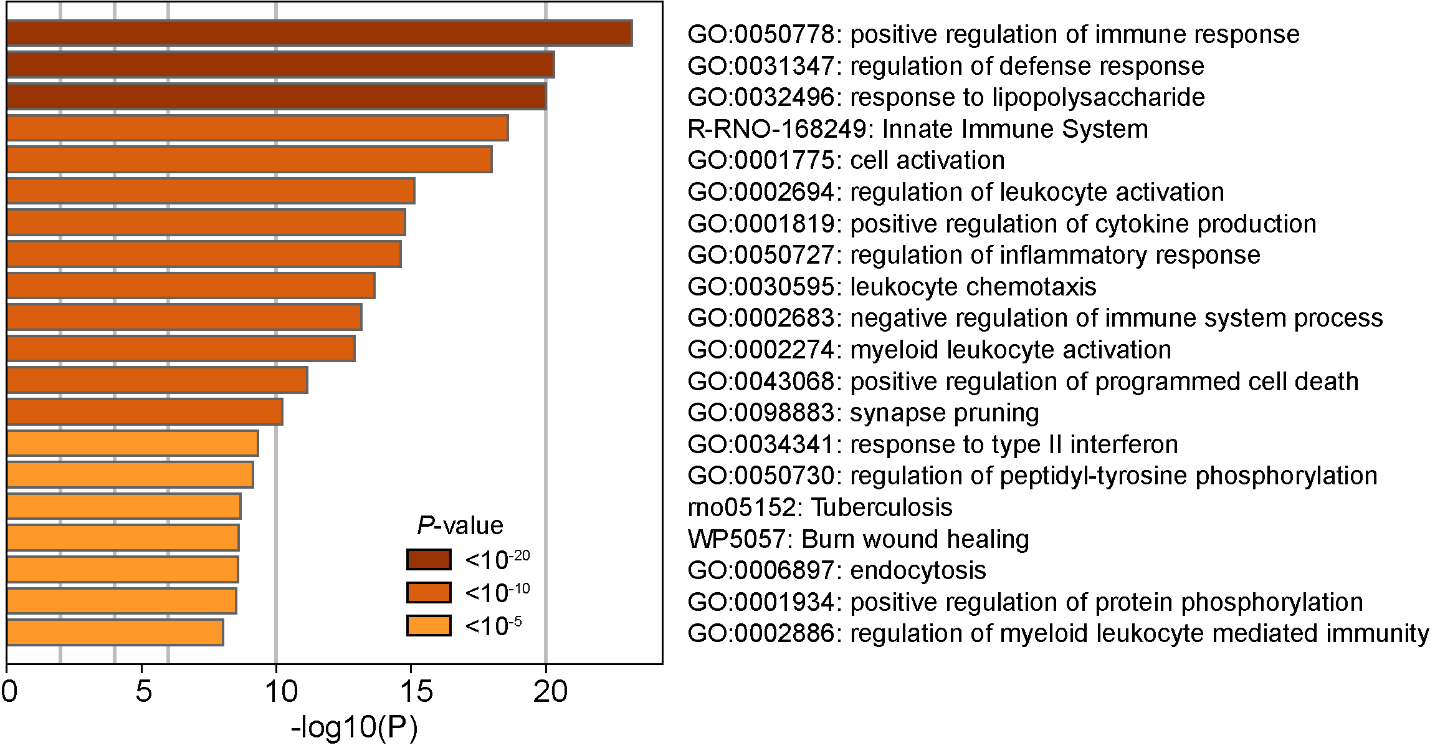


**Figure S5.** **Metascape bar graph for viewing top enrichment clusters.** Macrophage-selective genes were picked up from IAN DEGs data using the Harmonizome database, and these were bar-plotted using Metascape. The length of each bar represents –log_10_ (*P-*value). The left shows GO terms.


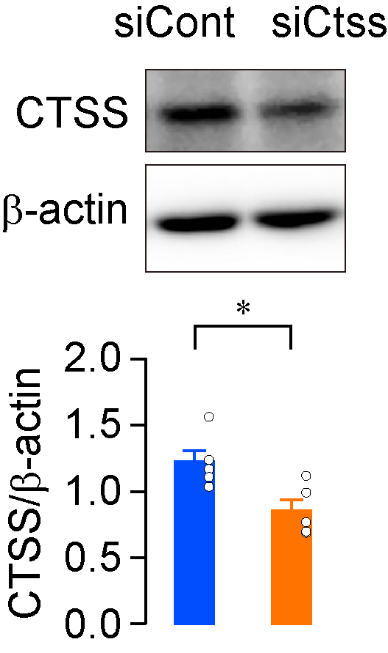


**Figure S6. Knockdown efficacy of CTSS after siRNA administration at the site of IAN injury post-IANX.** Blot of CTSS at the injured site 14 days post-IANX. siCont and siCtss indicate negative control siRNA and Ctss siRNA, respectively. The column represents the average values of CTSS/β-actin. n = 5 in each, unpaired *t*-test, **P* < 0.05. Data represent the mean ± SEM. All data points are shown in open circles.


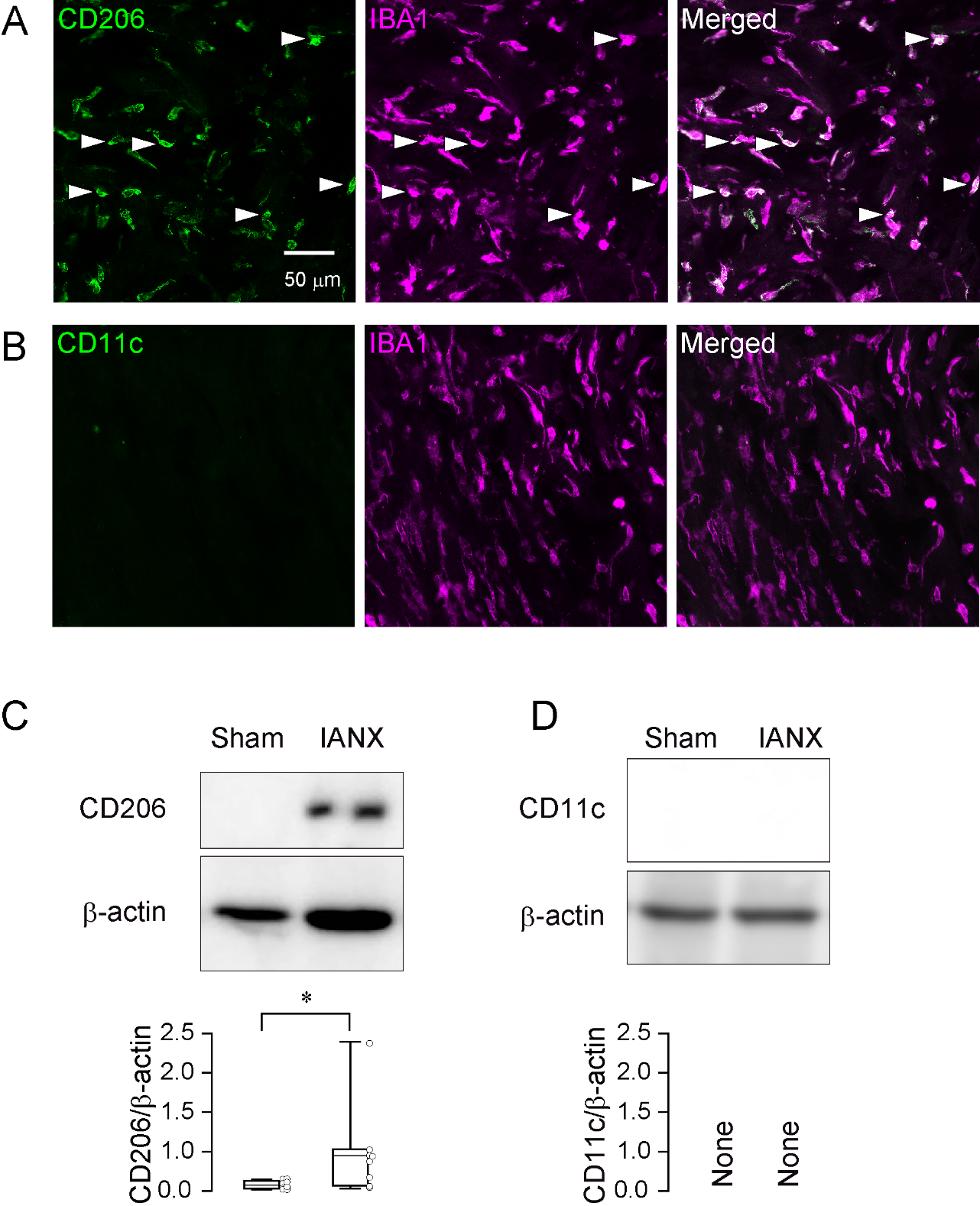


**Figure S7. Macrophage phenotype at the site of IAN injury. A, B** Images show CD206, CD11c, or IBA1 at the injured site 14 days post-IANX. Arrowheads indicate CD206 and IBA1 double-positive cells. **C, D** Blots show CD206 (C) and CD11c (D) at the injured site 14 days post-surgery. The column represents CD206/β-actin (C) or CD11c/β-actin (D). CD11c expression was undetectable at the injured site. n = 8 in each, Mann–Whitney U test, **P* < 0.05. Boxes show the 25th–75th percentiles with the median value as a line within each box, and whiskers indicate the 10th and 90th percentiles of the data in (C). All data points are shown in open circles.


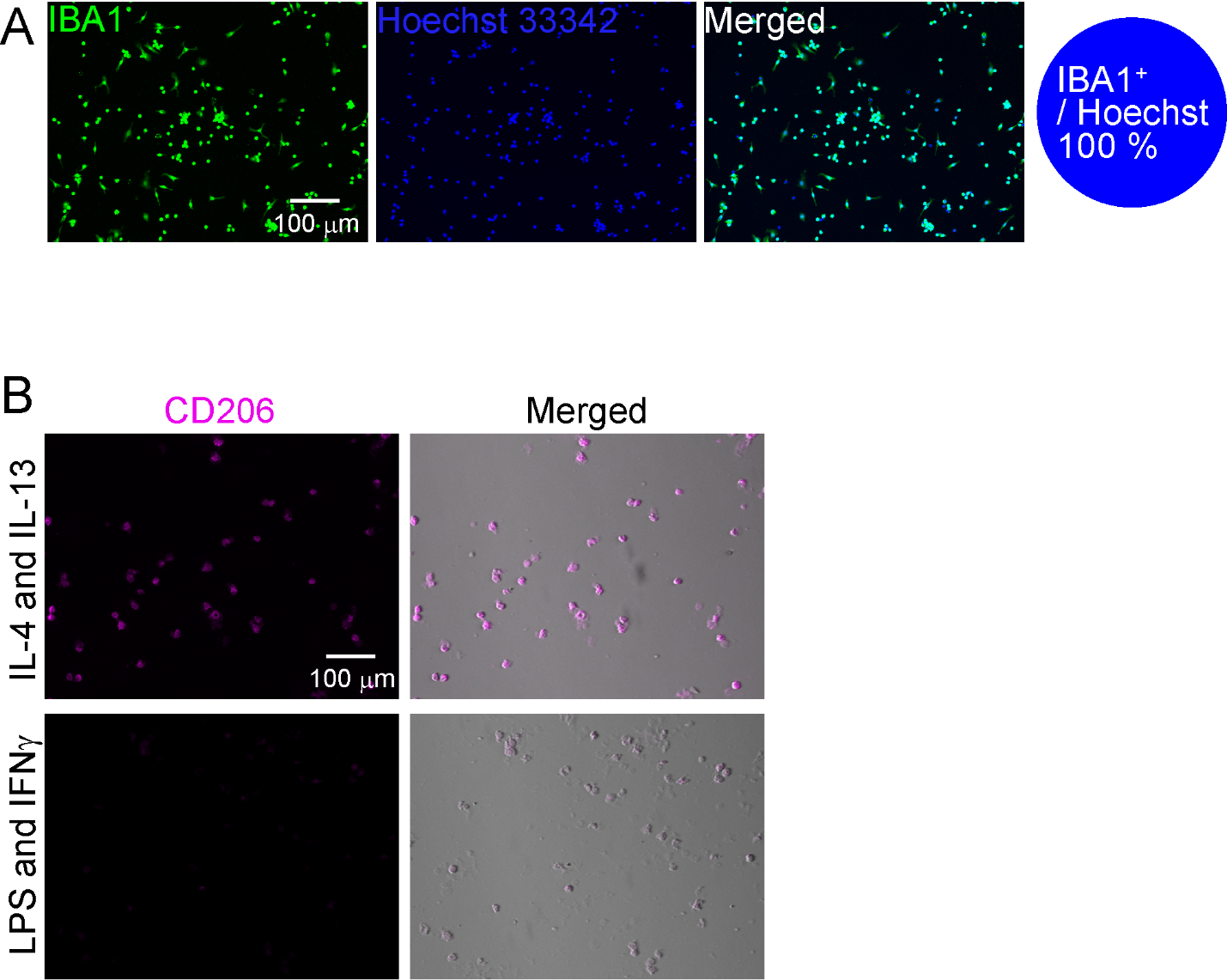


**Figure S8. Polarization of peritoneal macrophages. A** Peritoneal macrophages were seeded on the plate and then non-adhesive cells were washed out. The images show IBA1 and Hoechst of the adhesive cells in the culture plate. The pie chart indicates IBA-positive cells per Hoechst-positive cells. N = 10 independent culture. **B** Macrophages were stimulated with IL-4 and IL-13 or LPS and IFNγ. The images show CD206 and differential interference contrast of macrophages.


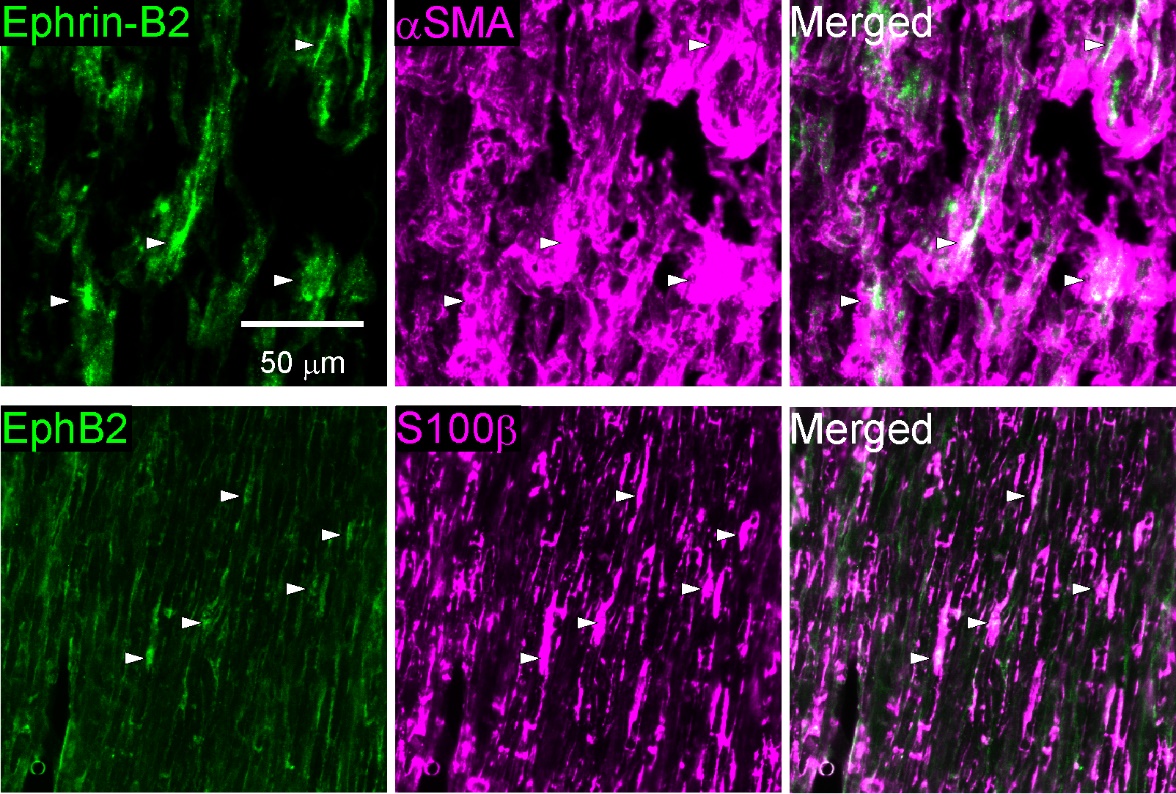


**Figure S9. Expression of Ephrin-B2 or EphB2 at the site of IAN injury 14 days post-IANX.** Images show Ephrin-B2, αSMA, EphB2, or S100β immunofluorescence at the injured site. Arrowheads indicate Ephrin-B2 and αSMA double-positive cells or EphB2 and S100β double-positive cells.


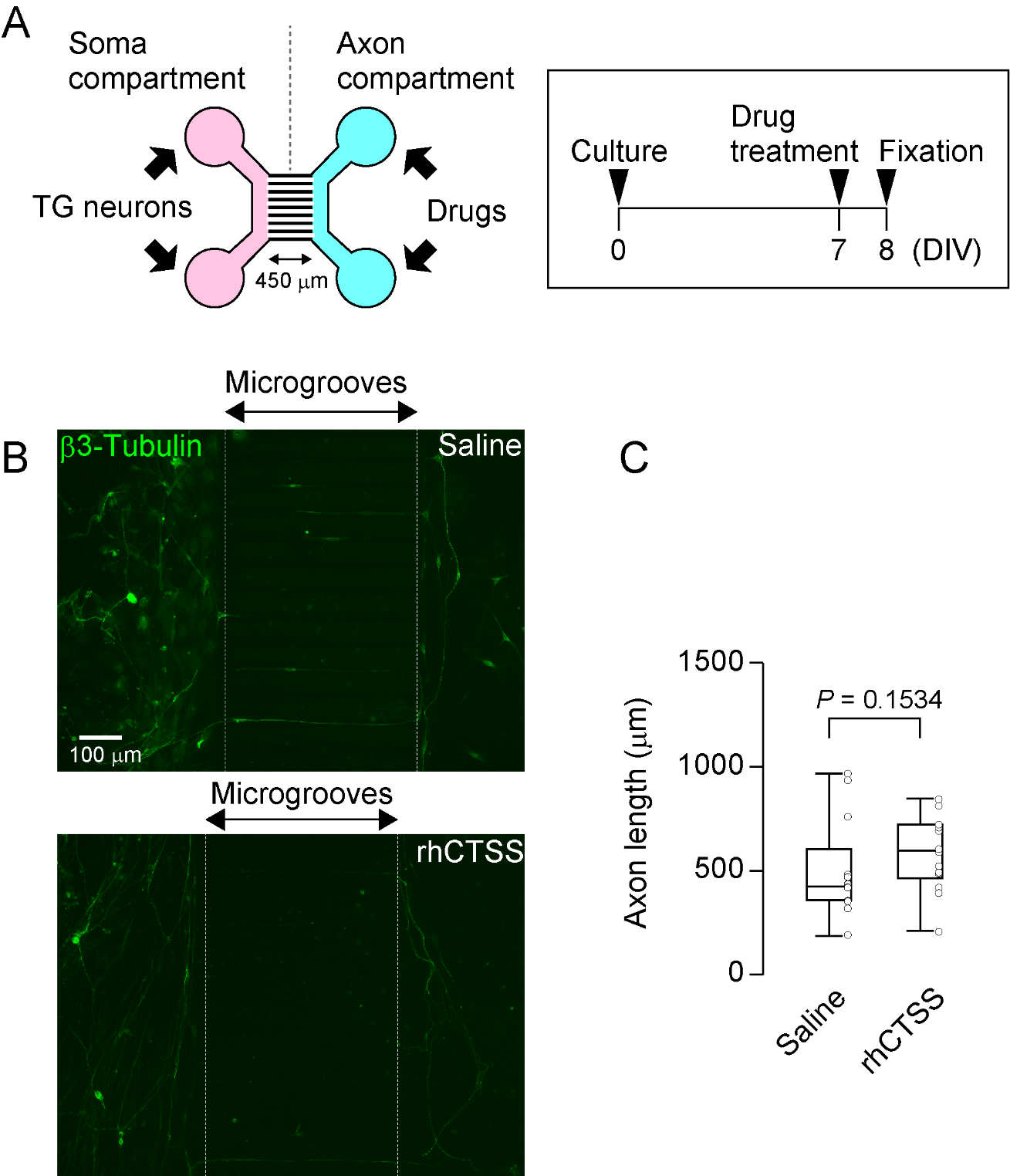


**Figure S10. Effects of rhCTSS on axon outgrowth in primary trigeminal ganglion (TG) neurons.** **A** Schematic illustration of a chamber of compartmentalized neuronal cultures and time course of the preparation. **B** Images show β3-Tublin immunofluorescence in primary TG neurons. The column represents the average length of axons after treatment with saline or rhCTSS. n = 13 independent cultures, Mann–Whitney U test, *P* = 0.1534. Boxes show the 25th–75th percentiles with the median value as a line within each box, and whiskers indicate the 10th and 90th percentiles of the data. All data points are shown in open circles.


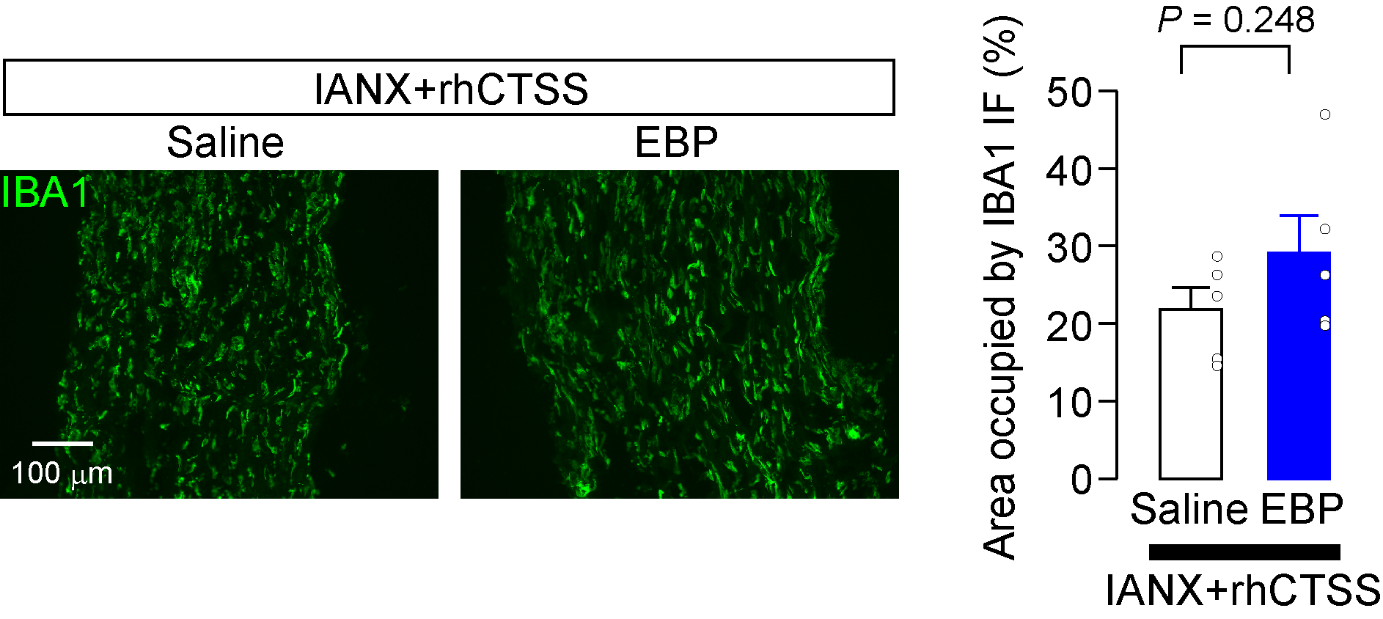


**Figure S11. Effects of Ephrin-B2 blocking peptide (EBP) on macrophage expression at the site of IAN injury 8 days post-IANX.** Images show IBA1-positive cells at the injured site. The column represents the average values of the area occupied by IBA1 immunofluorescence at the injured site. n = 5 in each, unpaired *t*-test, *P* = 0.248. All data points are shown in open circles.

**
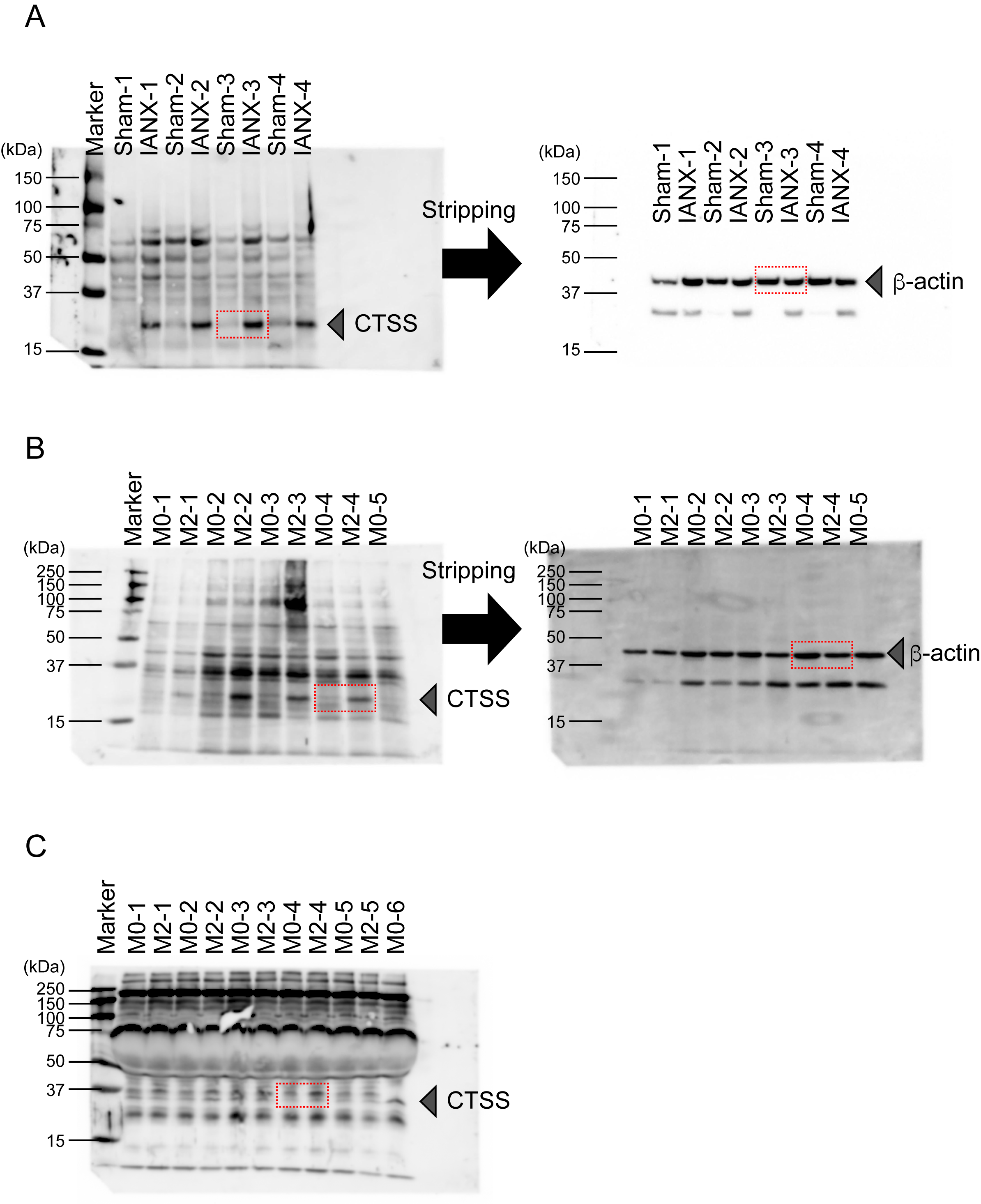
**

**Figure S12** Original blot for Figuer 2D (A), Figuer 3B (B), and Figure 3C (C). The area enclosed by the red broken line is the image used in the figure.

**
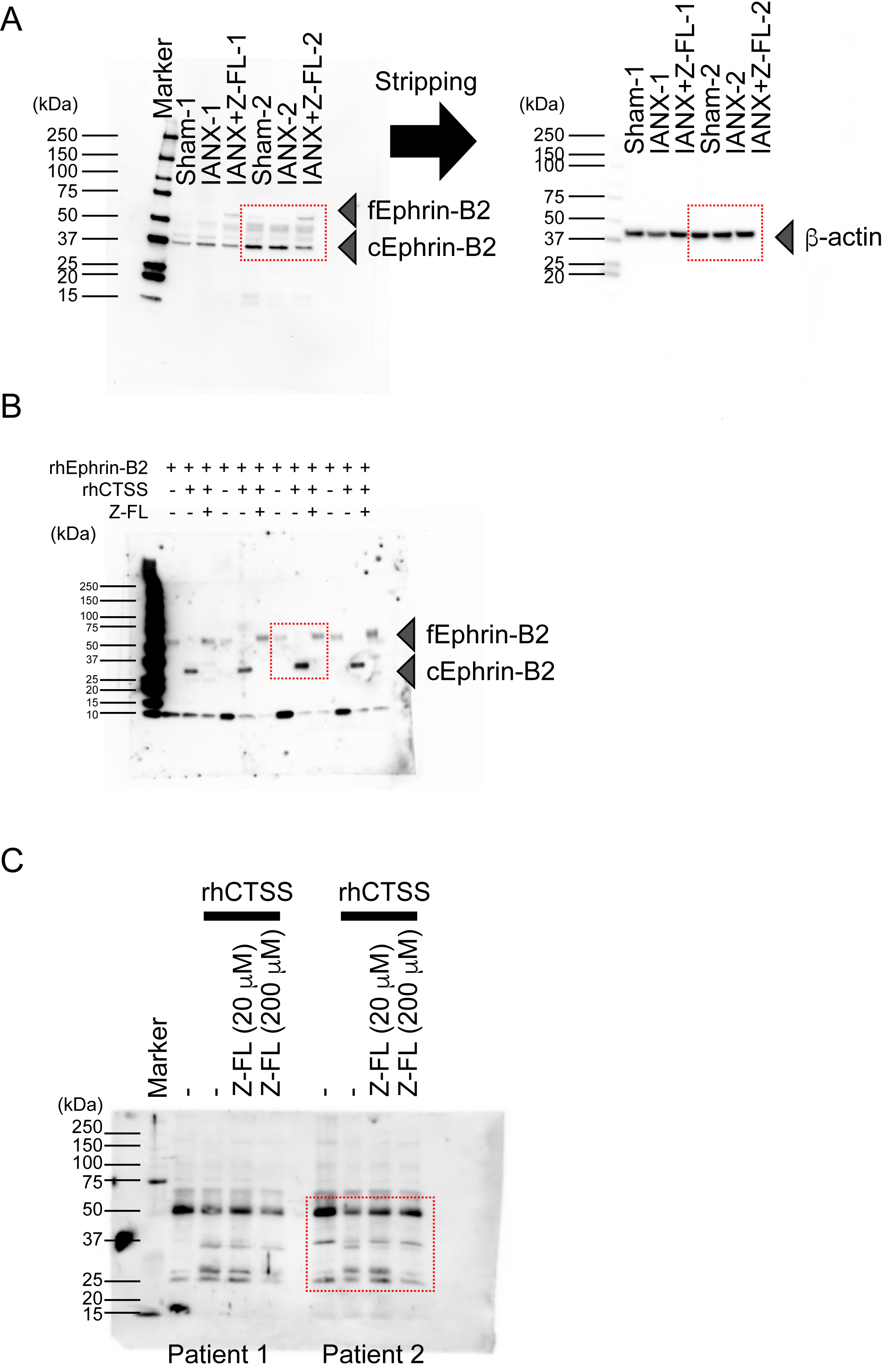
**

**Figure S13** Original blot for Figuer 4A (A), Figuer 4D (B), and Figure 5B (C). The area enclosed by the red broken line is the image used in the figure.

**
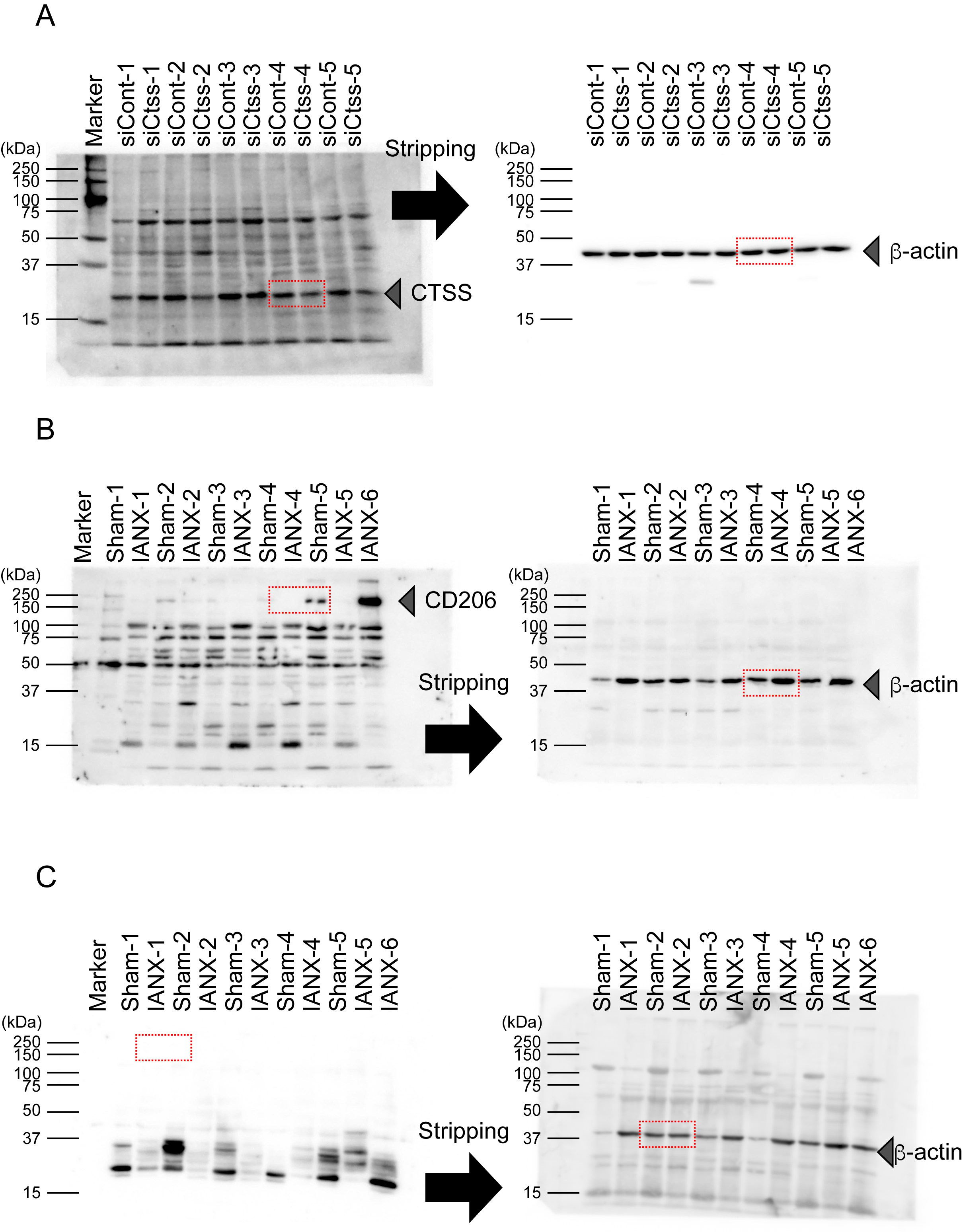
**

**Figure S14** Original blot for Figuer S6 (A), Figuer S7C (B), and Figure S7D (C). The area enclosed by the red broken line is the image used in the figure.
